# UMI-linked consensus sequencing enables phylogenetic analysis of directed evolution

**DOI:** 10.1038/s41467-020-19687-9

**Published:** 2020-11-26

**Authors:** Paul Jannis Zurek, Philipp Knyphausen, Katharina Neufeld, Ahir Pushpanath, Florian Hollfelder

**Affiliations:** 1 Department of Biochemistry, University of Cambridge, Cambridge, CB2 1GA UK; 2 Johnson Matthey Plc, Cambridge, CB4 0WE UK

**Keywords:** High-throughput screening, Next-generation sequencing, Synthetic biology, Biocatalysis

## Abstract

The success of protein evolution campaigns is strongly dependent on the sequence context in which mutations are introduced, stemming from pervasive non-additive interactions between a protein’s amino acids (‘intra-gene epistasis’). Our limited understanding of such epistasis hinders the correct prediction of the functional contributions and adaptive potential of mutations. Here we present a straightforward unique molecular identifier (UMI)-linked consensus sequencing workflow (UMIC-seq) that simplifies mapping of evolutionary trajectories based on full-length sequences. Attaching UMIs to gene variants allows accurate consensus generation for closely related genes with nanopore sequencing. We exemplify the utility of this approach by reconstructing the artificial phylogeny emerging in three rounds of directed evolution of an amine dehydrogenase biocatalyst via ultrahigh throughput droplet screening. Uniquely, we are able to identify lineages and their founding variant, as well as non-additive interactions between mutations within a full gene showing sign epistasis. Access to deep and accurate long reads will facilitate prediction of key beneficial mutations and adaptive potential based on in silico analysis of large sequence datasets.

## Abstract (web summary)

The success of protein evolution is dependent on the sequence context mutations are introduced into. Here the authors present UMIC-seq that allows consensus generation for closely related genes by using unique molecular identifiers linked to gene variants.

## Introduction

Directed evolution has emerged as a viable route to improve biocatalysts, as recognized by the Nobel Prize in Chemistry in 2018^1^, but governing the intrinsically random process of an evolution campaign to ensure its success is a daunting challenge. Evolution can be conceptualized as an exploration of “sequence space” along possible trajectories that lead to useful sequences with desirable properties^2,3^. The combination of ultrahigh throughput screening and next-generation sequencing is a powerful way of mapping such combinatorial explorations of protein function. The knowledge obtained may be used to steer experimentation into promising regions within the “vastness of sequence space”, simultaneously helping to understand fundamental concepts underlying sequence-function relationships^4–6^. Examples of this approach have revealed e.g. that epistasis is responsible for shaping the local fitness landscape of GFP^7^, provided insight into the trade-off between enzyme solubility and activity^8^ or enabled the structural prediction of different small protein binders^9,10^. Similarly, data parameterization starting from sequence information enabled the design of selective peptide binders using machine-learning approaches^11^. There are indications that gathering extensive sequence datasets during evolutionary transitions is crucial for the development of machine-learning algorithms to enable data-driven predictions for protein engineering based on statistical models of protein function^12–16^.

Evolutionary lineages are characterized by non-linear interactions between mutations within one gene that can only be inferred from accurate full-length sequence analysis of gene variants. Such “epistatic” interactions are key determinants of protein evolution and synergy or incompatibility of mutations determines which pathways are accessible in sequence space^17–19^. To delineate and understand the principles of these interactions, high throughput and accurate full-length sequences of gene variants are necessary. While Sanger sequencing can provide accurate sequence information with read lengths covering the extent of typical genes (0.9–1.4 kb)^20^, it is very cost-intensive when high throughput is required. Sequencing-by-synthesis platforms such as Illumina, on the other hand, offer great accuracy and throughput, but their primary readout is limited to short reads (up to 300 bp), falling far short of the average gene length. Illumina sequencing is thus unable to directly capture simultaneously occurring mutations across a full gene. This dilemma can be overcome with synthetic long reads, which are generated by associating multiple short reads into long sequences. However, synthetic long-read methods for amplicon sequencing are known to suffer from uneven coverage^21–23^ or high chimera rates^24,25^, resulting in a substantially reduced number of correct full-length sequences and thus effectively lowered throughput (for more details see “Discussion”). Third-generation single-molecule sequencing technologies (such as those developed by Pacific Biosciences and Oxford Nanopore Technologies) are able to generate truly long reads at high throughput but suffer from high error rates, preventing the accurate assignment of single point mutations to individual reads (e.g. for nanopore sequencing with an accuracy of 85-95% at least 50 in 1000 bases are assigned incorrectly)^26^. The extensive read lengths enable direct association of co-occurring mutations; however, generation of consensus sequences from multiple reads is necessary to increase accuracy.

Accurate consensus sequences are commonly generated for genome assemblies, where every sequencing read represents a unique fragment that overlaps with many others, facilitating stacking for accurate consensus generation^27,28^. By contrast, libraries in directed protein evolution comprise rather closely related members. Here the sequence diversity in randomized gene libraries is typically low: protein variants with a few or even a single point mutation can be functionally different. An erroneous raw third-generation sequencing read cannot reliably be assigned to its template, when multiple template candidates differing only in few point mutations are possible. This inability to assign reads to any one parental molecule therefore renders consensus-based “polishing” from unlinked reads impossible. Similar samples in which individual members differ merely in a few nucleotides can also be found in e.g. immune repertoires, metagenomic 16S amplicons or medical diagnostics, calling for a suitably high-quality sequencing solution. Current approaches to increase the accuracy of nanopore sequencing rely on the generation of concatemerized sequencing templates with multiple copies of the same gene variant forming a single DNA molecule. This physical copy-linkage results in long reads containing multiple “sub-reads” of the same gene variant, based on which a consensus sequence can be generated with up to 99.5% accuracy^29^ (i.e. 5 in 1000 bases are assigned incorrectly), depending on gene and concatemer length. However, this accuracy is still insufficient to distinguish point mutations and practically the workflow includes multiple delicate steps that require careful handling of high-molecular weight DNA molecules.

To achieve higher nanopore sequencing accuracy, we developed a workflow generating a high-quality consensus based on a bioinformatically traceable sequence link between descendant reads and their molecule of origin by introducing random DNA barcodes (i.e. unique molecular identifiers, UMIs) prior to amplification for sequencing. UMIs are widely used to retain information on true molecule numbers before PCR amplification. Specifically, the distinct template identity marked by the UMI enables confident identification and quantification of PCR duplicates^30^. This method has been applied e.g. to achieve unbiased counting of molecules for expression data in RNA-seq experiments^31,32^ and the accurate quantification of rare mutations^33,34^. Here, we leverage UMI-tags to assign erroneous nanopore reads to their molecule of origin, facilitating clustering for accurate consensus formation even when starting with a pool of highly similar sequences (e.g. a library of gene variants in protein evolution generated by error-prone PCR). Such sequences typically differ in only a few point mutations and can currently not be distinguished reliably in an ordinary nanopore sequencing output. We apply our workflow to protein engineering and demonstrate the analysis of high-quality full sequence outputs through rounds of ultrahigh throughput directed evolution of an amine dehydrogenase (AmDH), tracking the emerging phylogeny—or the “walk through sequence space”^2^—towards higher activity in directed protein evolution.

## Results

### UMIC-seq is a nanopore sequencing workflow for libraries with few nucleotide variations

**Fig. 1:**
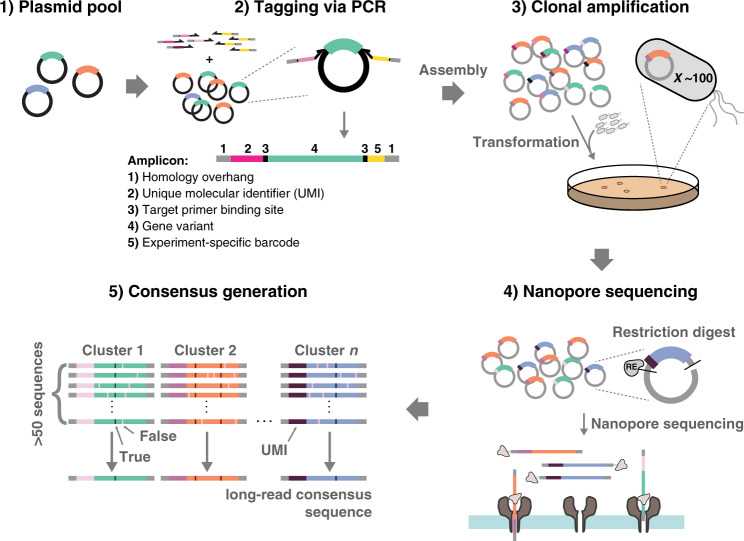
Sequencing strategy for amplicon libraries with UMI-linked nanopore long reads (UMIC-seq). An amplicon library with similar members, such as a plasmid pool of improved enzyme variants, an immune repertoire or metagenomic 16S sequences (1) is used as input to UMI-tagging and barcoding by PCR (2). The resulting products have unique sequence identifiers (UMI) for clustering and barcodes for multiplexing, as well as homology overhangs used for Gibson assembly. Gibson assembly provides plasmids for transformation, which enables the selection of a subset of the library, obtaining selectively amplified UMI-variant combinations (3). This sample is then subjected to nanopore sequencing (4) and data processing via barcode- and UMI-based clustering followed by variant calling, yielding the desired variant sequences with high accuracy (5).

To achieve easy control over molecule count and consensus coverage every library member was tagged with a unique molecular identifier (UMI) (Fig. 1). The UMIs allow individual sequencing reads to be assigned to their template molecule, enabling the generation of UMI-linked consensus sequences with very high final accuracy (>99.99%). Specifically, we increased sequencing accuracy by tagging and selectively amplifying single molecules with UMIs, which enable the generation of consensus sequences from many reads per variant. The UMIs have to be highly diverse to allow faithful assignment of raw, low-accuracy nanopore reads to the corresponding variant. We have performed simulations of clustering efficiencies using our UMIC-seq workflow under different error-rate regimes and library sizes to estimate suitable UMI lengths for our application (Supplementary Fig. 1). Based on these simulations, fully randomized 50 bp sequences were chosen as UMIs for subsequent experiments, as they were predicted to enable complete recollection of clusters over a broad range of error rates (Supplementary Fig. 1a) as well as homogeneous clusters in libraries of up to a million variants (Supplementary Fig. 1b). The UMIs were incorporated using primers in two PCR cycles, with deliberately low amplification so that potential PCR bias was reduced compared to other methods.

As the number of theoretically possible UMI sequences (4^50^) far outweighs the number of template molecules in the PCR reaction, each template molecule will be uniquely tagged. The number of UMI-tagged molecules generated, however, is too large to achieve deep sequencing coverage. It is thus necessary to selectively amplify a small subset of UMI-variant combinations. Uniquely, the UMI-variant complexity is reduced by a transformation step into cells, because an exact number of molecules is represented in the number of transformant colonies, directly combining selective amplification and control over molecule count in a straightforward molecular biology step. No PCR other than the two cycles of UMI-tagging are strictly necessary for DNA amplification, because transformed molecules will be amplified within the cell. This step minimizes bias compared to the alternative of PCR amplification of an initially small number of template molecules. It would be possible to dilute the initial pool of variants (linked to many different UMIs) to obtain the desired molecule copy number for a suitably reduced subset. However, control over the actual copy number is difficult to achieve experimentally (being dependent on accurate DNA quantification) and amplification of templates present in low copy numbers has been shown to be a major source of bias due to PCR stochasticity^35^. The transformation step thus combines simplicity with avoidance of bias in providing a defined number of variants from a large pool of molecules, easily identifiable by the colony count after transformation. Next, DNA is straightforwardly isolated from individual colonies and sequenced using current standard nanopore amplicon protocols. During data processing, the experiment-specific barcodes were used to de-multiplex sequences according to their selection round and the UMI-tags enabled clustering reads of different template molecules. Clustering was performed with a fast, “greedy” agglomerative algorithm (see our scripts available at https://github.com/fhlab/UMIC-seq) due to the immense number of sequence comparisons that need to be performed, limiting the use of most conventional all-vs-all comparisons for clustering. Potential mutations are identified for each cluster via signal-level analysis with nanopolish^27^, using the parental gene sequence as a reference. Additionally, filtering based on a nanopolish score of each potential mutation allows a distinction between false positive mutations (mainly insertion and deletion errors) and true mutations (mainly substitutions). In fact, filtering mutations based on a read support fraction of greater than 60% deplete errors that consistently occur in one of the two read directions (Supplementary Fig. 2a), as also shown by a similar filtering approach provided by a recent study in a different context^36^. With this filtering threshold, and using 180 pairs of Sanger reads (a total of 173 kb) as reference, we calculate a mean per-base error rate of 0.008% from as little as 35-fold sequencing coverage (Supplementary Fig. 2b). We identified 654 true mutations via Sanger sequencing of the 180 reference variants, of which 98% were correctly identified in the UMIC-seq workflow. The mean error rate per mutation (or single-nucleotide variation) for the nanopore consensus sequences were 1.99% false negatives and 0.16% false positives. The relatively higher number of false negatives is likely due to errors in clustering, resulting in mixed clusters for which no mutations are called (Supplementary Note 1). The resulting slight inflation of WT-like non-mutated sequences in the dataset does not interfere with the analysis of phylogeny and trajectory in protein evolution.

### Accurate long-read sequencing applied to the microfluidic evolution of an amine dehydrogenase

**Fig. 2:**
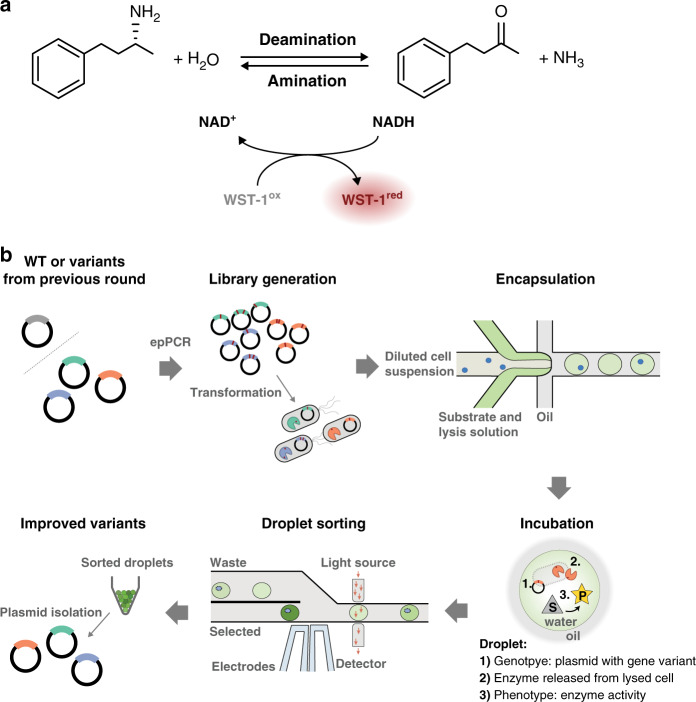
Ultrahigh-throughput droplet microfluidic evolution of an amine dehydrogenase (AmDH) biocatalyst. **a** Coupled reaction to measure AmDH activity in droplets. The enzyme AmDH catalyzes the deamination of (*R*)-1-methyl-3-phenylpropylamine to 4-phenyl-2-butanone, thereby reducing NAD^+^ to NADH and releasing NH_3_. NADH is regenerated upon reduction of WST-1, mediated by 1-methoxy-5-methylphenazinium methyl sulfate (mPMS). Reduced WST-1 exhibits absorbance at 455 nm, which is detectable by absorbance-activated droplet sorting (AADS) on chip^38^. **b** A cycle of directed evolution using absorbance-activated droplet sorting (AADS). Generation of diversity by error-prone PCR was followed by transformation and compartmentalization of cells in droplets. Cell lysis upon droplet formation liberates AmDH and reaction progress was determined based on the formation of colored downstream product in a coupled reaction. The best ~0.5% of variants in the library were selected by AADS and the respective plasmid pool was isolated.

To exemplify the utility of the UMIC-seq workflow, we applied directed evolution to an AmDH (Fig. 2), a valuable biocatalyst for the synthesis of chiral amines^37^. This enzyme can be screened in an ultrahigh throughput droplet microfluidic assay (>10^5^ droplets per hour)^38^, which leads to a large harvested hit diversity exceeding the limits of conventional hit analysis based on Sanger sequencing. Our objective was to track the accumulation of mutations in pools of variants and retain information on the co-occurrence of mutations over three rounds of directed evolution (Supplementary Fig. 3). Specifically, 1-3 mutations were introduced per gene and per round of directed evolution using error-prone PCR of the parental AmDH sequence (in the first round) and the obtained hit pools (for the consecutive rounds). The respective enzymes were expressed in *E. coli* that were compartmentalized in droplets as single cells, where lysis agent liberated the expressed enzyme and encounter with the reaction substrate allowed the enzymatic reaction to proceed. Product formation could then be detected as an optical read-out (Fig. 2). In each round of screening, the 1000 best hits out of ~250,000 variants were isolated via absorbance-activated droplet sorting^38^ (AADS, Supplementary Fig. 4) and sequenced using the UMI-linked long-read workflow with an Oxford Nanopore MinION flow cell.

### Long-read sequencing information uniquely informs protein engineering

**Fig. 3:**
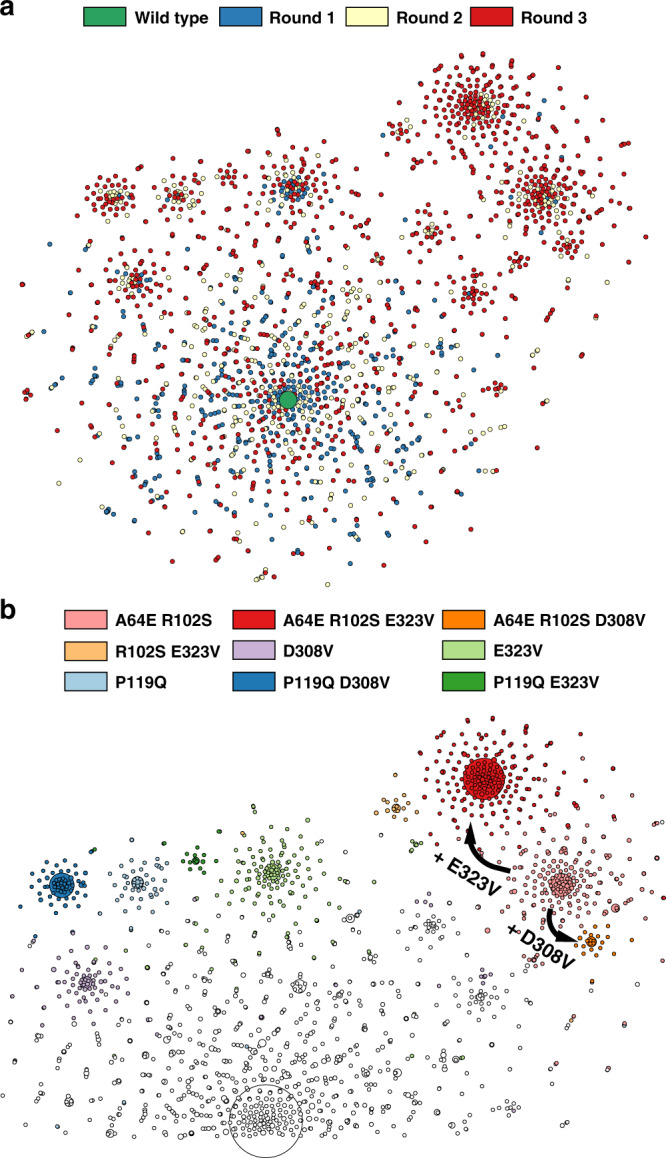
Phylogenetic analysis of directed evolution in sequence similarity networks. **a** Full graph color coded by the round of evolution in which mutant sequences were first recorded. A graphical representation (with t-Distributed Stochastic Neighbour Embedding, tSNE) of variants identified in droplet screening illustrates possible trajectories in a fitness landscape: spots represent all unique variants identified within the pools of hits. Distinct clusters emerge over the course of evolution, as indicated by the color code showing the round of first emergence of each sequence. Size of wild type increased for visualization. **b** Analysis of founder variants. Part of the sequence similarity network is shown with the color indicating the corresponding core set of mutations that they share with the ‘founder variant’, so that these mutations are present in all variants within the cluster. Spot sizes correspond to total sequence count of the respective variant. The clusters are defined by multiple mutations and thus can only be inferred based on long sequence reads that inform phylogeny of possible trajectories during experimental directed evolution.

**Fig. 4:**
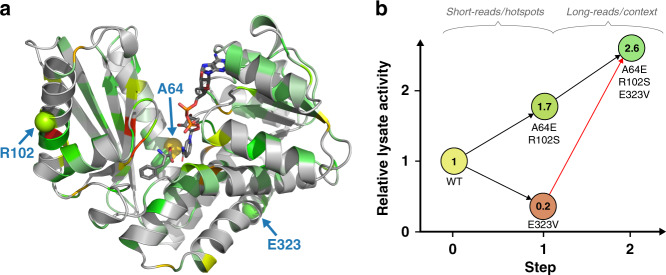
Analysis of epistasis in variant A64E R102S E323V. **a** Hot-spot analysis. Positional enrichments mapped onto the structure of a homologous AmDH (PDB ID: 1C1D). If the frequency of mutation at a certain position increased over the rounds of directed evolution, its enrichment factor is color coded on the structure from green (low) to yellow to red (high). Enrichment was calculated from round 1 to round 2 for variants that persist into round 3. Positions at which mutations are enriched can be identified over the full range of the enzyme, with many enriched positions e.g. in the loop covering the substrate binding pocket. Positions exhibiting epistatic interaction (variant A64E R102S E323V) are highlighted. **b** Sign epistasis in variant A64E R102S E323V detected by long-read sequencing. Graph showing lysate deamination activities (initial rates) of constituent variants relative to the non-mutated parent. Sign epistasis can be seen in mutations of one founder variant (A64E R102S E323V). The mutation E323V individually decreases activity drastically (16% of the parental AmDH (WT) lysate deamination activity, Supplementary Fig. 5), while it has a beneficial impact when introduced into the variant A64E R102S (154% of A64E R102S lysate deamination activity). With conventional short-read sequencing for hot-spot analysis, E323V would have wrongly been determined as an activating mutation; yet E323V is only beneficial in the context of other mutations, which was correctly detected by long-read sequencing.

A hot-spot analysis based on the frequency of mutation during the experimental directed evolution not only revealed several positions randomly scattered over the full length of the protein, but also indicated accumulation close to the loop covering the substrate binding pocket (Fig. 4a). This underlines the importance of less obvious distal mutations to protein function^39,40^. Hot-spot analysis based on evolutionary conservation from established sequence data is often used as guidance for site-directed protein engineering^41^; however, a hot-spot analysis does not report on the compatibility of the hot-spots with each other and with mutations in the rest of the protein. In contrast to hot-spot analysis, the sequence information obtained in this workflow describes multiple mutations in each variant over the full length of the gene (Supplementary Fig. 3): now improved variants with multiple mutations can be identified. Consequently, epistatic interactions, which are prevalent in protein evolution and difficult or impossible to predict^17,18^, can be detected and the possible trajectories can be visualized in a sequence similarity graph (Fig. 3). The sequence similarity graph clusters the sequences of mutants selected in each round of evolution into a phylogenetic structure based on the distance of its constituents in sequence space. In later rounds of evolution, variants start to cluster and evidently form lineages (Fig. 3a). Interestingly, “founder variants” of emerging clusters can be identified (Fig. 3b): in this context they are defined as the shared set of mutations present in all variants within a cluster. Closely related sequences cluster and share the core set of mutations of the “founder variant”. Even within three rounds of directed evolution, steps to form sub-lineages can be identified, e.g. the acquisition of mutations E323V or D308V to previous founder variant A64E R102S.

The abundance of each of these central motifs increases over the rounds of directed evolution. We assume that the founder variants bear one or more beneficial mutations, thus being selected more frequently in the droplet microfluidic assay. As a result, the abundance of this sequence motif increases in the pool and also produces a large offspring of related sequences after the next round of diversification and selection. To verify this hypothesis, we set out to test the activity of all founder variants. Additionally, epistatically interacting mutations can be derived in this workflow based on information about co-occurrence of mutations and the knowledge of phylogenetic lineages helps to overcome epistatic limits that protein engineers often encounter^18^. This led us to not only test the founder variants alone, but also test all combinations of constituent mutations of the founder variants for non-additive effects.

Because a pool of variants is selected in the microfluidic screen and subsequently sequenced, individual variants are practically not easily retained and experimentally tested. Thus, variants were regenerated: point mutated primers were incorporated in whole-plasmid PCR, in a straightforward cloning strategy termed IVA cloning^42^. This procedure provided quick access to experimental testing of the core sets of mutations. However, for variants that contain a larger number of mutations a reconstruction from the wild-type sequence might be rather labor-intensive. Here, the UMI obtained in the sequencing effort can be used to fish out any unique mutant from the physical DNA pool of variants. Practically, such a dial-out PCR produces the variant of interest from the UMI-tagged sequencing pool^43^: using a UMI-specific primer this variant of interest is selectively amplified and cloned into an expression vector.

All mutations in the core sets were tested individually and in combination in a lysate activity assay (Supplementary Fig. 5). A lysate activity assay was chosen at this point because it resembles the selection conditions in droplets more closely than a purified protein assay. Expression level and activity are linked in a lysate assay similar to the conditions in the single-cell lysate droplet assay, where a function of both variables is responsible for product formation.

Interestingly, an example of sign epistasis can be seen in the founder variants A64E R102S E323V. The mutation E323V individually decreases lysate deamination activity to 15.7 ± 1.1% (95% CI) of the non-mutated parent, but it has a beneficial impact in the background of the founder variant A64E R102S, increasing its lysate deamination activity to 154 ± 33% (95% CI) of the A64S R102S variant (Fig. 4b). The basis of the interaction of these three mutations is not obvious, as they are far apart (Fig. 4a highlights the distance of at least 18 Å between the mutations that are shown to interact epistatically). The identification of such functionally relevant long-range interactions is only possible when long sequence reads are available: in our case the epistatic interaction extends across 780 bp (or 260 amino acids) in the sequence. This distance exceeds the conventional length limitations of second-generation sequencing that can only be overcome by assembling synthetic long reads in silico (see “Discussion”). To the best of our knowledge, the relationship displayed in Fig. 4 is the longest-ranging epistatic interaction in a protein identified in a high throughput mapping experiment to date. Conventional hot-spot analysis based on short-read sequencing (i.e. analyses such as the study of glycosidase catalysis via microfluidic deep mutational scanning)^44^, might have highlighted important residues but would have annotated all high-frequency mutations as activating. Yet E323V is only activating in the context of mutations A64E and R102S.

### Characterization of the founder variant A64E R102S E323V

The particularly successful founder variant A64E R102S E323V and the contributing mutation combinations were characterized with regard to their catalytic properties and performance as biocatalysts (Table 1). Interestingly, the mutation A64E has a strong contribution to an altered catalytic profile, increasing both the *K*_M_ and the *k*_cat_. The *k*_cat_ of variant A64E is almost 4-fold increased, indicating a strong selection for fast turnover at high substrate concentrations. This is in accordance with the directed evolution principle “you get what you screen for”^45^, since the absorbance detection in droplets requires high substrate turnover^38^. Thus, the droplet screening assay is performed with long incubation times at high substrate concentration, putting selection pressure on high *k*_cat_ values. Additionally, mutation A64E increases expression strength which is an important factor in lysate assays, such as the droplet microfluidic screening, as it increases the amount of available protein in the compartment. Mutation E323V, on the other hand, individually is deleterious in the lysate assay (Fig. 4b), stemming from a reduced catalytic efficiency as well as a stark reduction in expression (Table 1). The decrease in protein expression caused by mutation E323V, however, is compensated by mutation A64E, laying the foundation of the epistatic interactions. When E323V is combined with the other mutations, a decreased *K*_*M*_ value results in higher catalytic efficiencies compared to mutation A64E alone, further increasing the overall performance of the combined variant in the lysate activity assay.

**Table 1:** Characterization of A64E R102S E323V core set of mutations.

Variant	Steady state kinetics^a^			T_m_^b^ (°C)	Soluble expression^c^ (%)	Total turnover number^d^
	*k*_cat_ (s^−1^)	*K*_*M*_ (mM)	*k*_cat_/*K*_M_ (s^−1^ mM^−1^)			
WT	1.55 ± 0.02	0.40 ± 0.02	3.84 ± 0.06	50.5 ± 0.17	97 ± 0.25	20,100 ± 1,180
A64E	6.12 ± 0.05	2.17 ± 0.06	2.83 ± 0.03	50.4 ± 0.08	99 ± 0.40	57,400 ± 730
R102S	1.72 ± 0.03	0.33 ± 0.03	5.27 ± 0.08	49.7 ± 0.09	96 ± 0.11	22,000 ± 330
E323V	1.20 ± 0.01	0.37 ± 0.02	3.28 ± 0.05	51.0 ± 0.09	79 ± 3.34	16,400 ± 700
A64E R102S	5.38 ± 0.07	1.86 ± 0.07	2.90 ± 0.04	48.1 ± 0.08	97 ± 0.22	51,500 ± 720
A64E E323V	4.94 ± 0.09	1.62 ± 0.09	3.05 ± 0.06	49.9 ± 0.05	94 ± 0.47	66,300 ± 2,990
R102S E323V	1.36 ± 0.02	0.38 ± 0.02	3.62 ± 0.05	49.9 ± 0.07	98 ± 0.18	16,200 ± 2,030
A64E R102S E323V	4.23 ± 0.07	1.27 ± 0.07	3.34 ± 0.06	48.9 ± 0.09	98 ± 1.45	65,900 ± 380

Source data are provided as a Source Data file. All values are given with standard deviations from three independent technical replicates.

^a^*Steady state kinetics*: Buffer: 100 mM Glycine-KOH pH 10. Co-substrate: 2.5 mM NAD^+^. Temperature: 22 °C. Substrate: *R*-1-methyl-3-phenylpropylamine 0 mM to 12.8 mM. Supplementary Fig. 6.

^b^*Melting temperature*: Differential scanning fluorimetry with SYPRO orange. Supplementary Fig. 7.

^c^*Soluble expression*: Protein expression performed at 20°C for 16 h. Soluble fraction compared to pellet via gel densitometry.

^d^*Total turnover number*: Small scale transformation with 50 mM 4-phenyl-2-butanone and 0.1 µM enzyme. Reaction performed at 30 °C for 72 h with glucose dehydrogenase cofactor recycling.

This increased performance is confirmed in a biocatalytic assay, the determination of the enzyme’s total turnover number. The total turnover number was determined in the industrially relevant amination reaction direction at high substrate concentration. Here, the variant A64E R102S E323V again outperforms not only the wild type but also mutation A64E by a wide margin, indicating the suitability of the proposed approach to protein engineering to solve an applied biocatalytic challenge.

While the strongly activating mutation A64E would have been found by summing up mutation frequencies in short reads, the compatibility and the enhancing effects of other residues would have been missed in a hot-spot analysis. Consequently, third-generation reads of entire genes (typically 0.9–1.4 kb)^20^ are necessary in order to discover complete sets of epistatic interactions in proteins. Enabling such long reads opens the door to resolving complex interaction networks that will help to predict protein structures^9,10^, train machine-learning algorithms for protein engineering^11,13,14,16^ or study the fundamental constraints of protein evolution^7,46,47^. The comprehensive investigation of such phenomena by the simple nanopore sequencing workflow of Fig. 1 will allow the discovery of patterns typical for short- and long-range epistasis, providing a valuable thesaurus of information for future protein engineering.

## Discussion

The sequencing workflow presented here provides highly accurate long-read sequences suitable for the analysis of entire protein engineering datasets with single-base precision at unprecedented low cost. With a MinION flow cell capacity of 10 Gb and assuming a gene of interest of 2 kb length, our workflow can generate approximately 100,000 accurate consensus sequences at 50-fold coverage. This entails a cost per mutant sequence of <1.1¢, a 450-fold reduction compared to around 5 USD using classical Sanger sequencing (Supplementary Note 2), with virtually no limit to gene lengths that can be analyzed.

Using regular Illumina short reads, genes have to be split up into fragments and association between random mutations over a full gene length are lost. The longest possible DNA stretch that can be sequenced straightforwardly with second-generation technology is 600 bp, obtained by associating forward and reverse sequencing reads^48^. To overcome these limitations, synthetic long reads are constructed from short reads by linking them via compartmentalized barcoding strategies, which has been commercialized e.g. by Moleculo or 10x Genomics. However, while these methods are suitable for genomics, they do not meet the challenges of amplicon sequencing. In plate-based methods fragments are pooled, making it unsuitable for libraries of highly similar variants^49,50^, whereas microfluidic droplet-compartmentalized barcoding would require a reduction in fragment input concentration to maintain the monoclonality of droplet compartments (i.e. reduce collision of multiple variants per droplet), thus limiting the throughput of the method^51^. Current approaches designed for one-pot synthetic long-read amplicon sequencing are limited in maximal fragment length by the short-read bridge amplification requirements^48,52^, have high numbers of chimeric reads^24,25^ or a low percentage of full length reads^21–23^. Uneven coverage over the full length of the parent fragment results in these methods needing high sequencing coverage and still often failing at generating full-length consensus sequences.

These issues are reflected in current investigations of pairwise epistatic relationships in proteins. These studies focus, in most cases, on short domains (e.g. 56 amino acids in the IgG-binding domain of protein G^53^, 37 amino acids in the WW domain of Yap1^54^, 32 amino acids at the interface of Fos and Jun^46^ or 25 amino acid blocks in the second RRM domain of Pab1^55^). Capturing the *entire* protein sequence would be necessary to reveal the complete, presumably more complex epistatic interactions in entire proteins rather than domains. The longest stretch of sequence analyzed for multiple simultaneous mutations in a combinatorial experiment are the 238 amino acids of GFP, that were analyzed to describe its fitness landscape^7^. Here, shorter primary reads were barcoded by a stepwise UMI duplication via restriction and ligation. This enabled the analysis of mutational impact over the full GFP sequence, but multiple restriction and ligation steps render the protocol difficult and any imperfection (e.g. chimera formation in the ligation step, mutations in the restriction sites in the gene) scale badly with longer gene lengths, requiring more of those steps which might distort the sequence readout quantitatively and qualitatively.

A straightforward single read over the entire gene length would remove these ambiguities: the long primary nanopore sequencing readout does not need a processing assembly, but a simple comparison to their parent variant is sufficient. UMIC-seq is a more reliable and practically easier method in our opinion and results in a simple and scalable workflow, robust with regard to read coverage. By using true long reads, the workflow is the same for all gene lengths and the coverage over the length of the gene is uniform. Practically, the third-generation sequencing method we present here is easier to implement than any other: starting from a simple plasmid stock, the tagged sequencing library is obtained based on straightforward steps that only involve Gibson assembly and one PCR. Only a benchtop nanopore sequencer is required and the entire procedure can be carried out by one laboratory operator, who has direct control over the number of consensus reads (or UMIs) generated, as defined by the colony count. (All scripts and protocols can be found in the Supplementary Information and at https://github.com/fhlab/UMIC-seq).

We expect further applications of UMIC-seq beyond improvement of biocatalysts to arise e.g. in long-read deep mutational scanning, sequencing of immune repertoires and metagenomic samples or medical diagnostics. Applications involving larger sequence deviation between variants, such as metagenomics, might require the assembly of a reference sequence prior to polishing, which can be easily implemented in the pipeline with tools such as Miniasm^56^ and Racon^28^. Additionally, deep mutational scanning (DMS) could seamlessly be integrated with UMIC-seq. DMS relates information on gene frequency changes to protein function for large scale studies of sequence-function relationships^6,57^. Currently, DMS is based on short reads, enabling the analysis of comprehensive single-site saturation libraries^58,59^ or multi-point mutants in short domains (as discussed above^46,53–55^). These large-scale multi-point mutagenesis datasets alone have recently been shown to enable the construction of small 3D structures^9,10^. However, determining mutational effects covering full gene lengths has been identified as a crucial next step in technology development towards larger structures^60^. With UMIC-seq accurate long reads become possible, so that multi-point mutations, even if far apart and randomly distributed across a whole gene, can be unearthed: studying functional epistasis in protein evolution in this way could simultaneously enable the mutagenesis-based determination of full protein structures. A synthesis of DMS and UMIC-seq would start with sequencing of the naïve library using the UMIC-seq workflow, resulting in accurate full-length sequences and their corresponding UMIs. In a second step, short-read sequencing of only the UMI-tag would suffice to determine the frequencies of variants after a functional assay, deriving fitness scores that quantify the (currently unpredictable)^17,18^ interactions of simultaneously occurring mutations throughout the full gene length of many variants.

Large numbers of long reads, obtained two orders of magnitude cheaper and with higher accuracy than possible at present, will powerfully inform emerging sequence-function relationships. This high information content will propel conventional phylogeny-based approaches, but also enable more advanced machine-learning techniques^61^ that predict cooperative fitness effects arising from intra-gene epistasis. The combination of easy-to-generate, large and low-cost datasets will make the exploration of sequence space for the analysis and engineering of functional proteins more accessible and likely more successful.

## Methods

### Reagents

All chemicals and oligonucleotides were purchased from Sigma-Aldrich unless otherwise noted. Enzymes were purchased from New England BioLabs. GDH-101 was provided by Johnson Matthey. Nanopore flow cells and kits were obtained from Oxford Nanopore Technologies. The plasmids pASK-IBA36b + and pRSF-Duet1 were purchased from IBA Lifesciences and Novagen, respectively.

### Mutagenesis

The amine dehydrogenase (AmDH, Supplementary Sequence 1) was cloned into pASK-IBA63b + at *Nco*I and *Xho*I sites and expressed in fusion with a C-terminal Strep-tag. A random mutagenesis library was generated by error-prone PCR, as described in the Gene-Morph II random mutagenesis kit manual (Agilent). A target concentration of 100 ng template (parental AmDH plasmid or plasmid pool) was amplified with the Mutazyme II low-fidelity polymerase for 25 cycles. The resulting PCR product was ligated into pASK-IBA63b + at *Nco*I and *Xho*I sites and used to transform E. cloni 10 G ELITE cells (Lucigen), yielding more than 10^7^ transformants at an average mutation rate of 3.5 mutations per gene. Individual variants chosen for characterization were generated with primers containing the desired point mutation, derived from sequencing and network building, by IVA cloning^42^. Point mutations were incorporated in the forward primer (Supplementary Table 1) for whole-plasmid PCR, in between a homologous recombination region (≥15 bp, *T*_m_ ≈ 50 °C) and a template binding region (*T*_m_ ≈ 60 °C). The reverse primer fit the homologous recombination region but was extended until a total T_m_ of 60 °C was reached. Plasmid containing the reference gene was used at 10 ng as template for 22 PCR cycles with the mutagenic primers, using Q5 polymerase according to manufacturer’s specification in a 25 µL reaction. Template DNA was removed by addition of 1 µL DpnI (FastDigest, Thermo Fisher) to the PCR reaction and incubation at 37 °C for 30 min. Finally, 1 µL of the unpurified digested PCR product was used to transform 25 µL chemically competent *E. coli* cells (NEB 5α high efficiency).

### Directed evolution by absorbance-activated droplet sorting (AADS)

*E. coli* cells were transformed with the AmDH library and grown in LB medium supplemented with 100 µg/mL ampicillin at 37 °C and 200 rpm. Protein expression was induced with 200 ng/mL anhydrotetracycline at an optical density at 600 nm (OD_600_) of 0.4–0.8 and cultivation continued overnight at 20 °C. The expression culture was washed with 100 mM glycine-KOH buffer (pH 9.0) and diluted in buffer containing 25% (*v/v*) Percoll to an OD_600_ of 0.01. Single cells were co-encapsulated in 280 pL droplets with substrate and lysis mix (10 mM WST-1 (NBS Biologicals), 10 mM (*R*)-1-methyl-3-phenylpropylamine, 2 mM NAD^+^, 5 µg/ml 1-methoxy-5-methylphenazinium methyl sulfate (mPMS), 1 µl/ml rLysozyme (Merck) and 0.8x CelLytic B in 100-mM glycine-KOH pH 9.0) using a flow-focusing chip of 80 µm height and 50 µm width (Supplementary Fig. 4). Droplet generation flow rates were 8 µL/min for the aqueous solutions and 30 µl/min for the oil phase (HFE-7500 (3 M Novec) containing 1% (*w/v*) 008-FluoroSurfactant (RAN Biotechnologies)). Droplets were incubated for 2 h at 22 °C to allow the reaction to proceed. After 2 h of incubation at 22 °C, droplets were injected in the sorting chip^38^ (Supplementary Fig. 4) at 100–120 Hz (2-3 µl droplet emulsion per min spaced by 30–40 µl/min HFE-7500) and sorted according to their absorbance at 455 nm. For each sample, a user defined threshold was set to select the best 1000 variants out of approximately one million droplets at 25% occupancy. Plasmid DNA from sorted droplets was recovered^62^ and used for the next round of mutagenesis and sorting, and as input for the nanopore sequencing.

### Sequencing library preparation

Sorted variants were the input for sequencing library preparation in form of pooled plasmid stocks. A detailed description of the sample preparation procedure is available (Supplementary Protocol 1)^63^. Briefly, PCRs were performed using Q5 High-Fidelity 2X Master Mix according to the manufacturer’s instructions. The forward primer contained an experiment specific barcode for each selection round (sequences taken from the PCR Barcoding Expansion Pack 1-96 (EXP-PBC096) to allow de-multiplexing) and the reverse primer contained two 25xN stretches (UMI); a total of 50 bp that allowed the formation of distinct clusters from erroneous raw reads after sequencing (Supplementary Fig. 1). Two cycles of PCR were performed to tag 500 ng input DNA with the UMI and the barcode. Tagged molecules were Gibson assembled into an acceptor plasmid (pRSF-Duet1, previously linearized with *Bam*HI and *Kpn*I) and a dilution series was plated after transformation. Each colony corresponds to one UMI-variant combination, which is clonally amplified by the growing cells. Plasmids from ~3000 colonies were isolated for each round of evolution, with the goal to generate as many accurate nanopore consensus sequences. Around 1000 unique variants are expected per round and 3000 colonies were chosen to account for oversampling of these variants. From this pool of amplified and UMI-tagged variants, the sequencing region was excised by restriction digest with *Bam*HI and *Kpn*I and sequenced with an ONT MinION equipped with a R9.4.1 flow cell after sample preparation according to the SQK-LSK109 sequencing library preparation kit. Sequencing was stopped once the sequencing read count reached 100-fold sample coverage: The expected ~9000 fragments of 1250 bp length required > 0.9 million reads to achieve 100-fold sequencing oversampling, corresponding to >1.1 Gb sequencing data.

### Derivation of consensus sequences

A workflow diagram showing the steps for bioinformatic analysis is shown in Supplementary Fig. S2. All scripts written as well as an exemplary analysis are made available at https://github.com/fhlab/UMIC-seq and stepwise explanations (Supplementary Protocol 2) are provided^63^. Briefly, basecalling was performed within the MinKNOW software as described in the SQK-LSK109 protocol. UMIs were extracted from the basecalled reads and used to cluster the obtained sequencing reads with a custom script. The clusters were aligned to the reference sequence and consensus sequences were generated with nanopolish^27^. The final data were filtered for quality control: variants with more than 16 mutations and mutations with a nanopolish support fraction lower than 0.6 were discarded.

### Enzyme activity assays

Lysate activity assays were performed in 96-well plates. Wells containing 400 µL LB medium (supplemented with 100 µg/mL ampicillin) were inoculated from single colonies and grown overnight at 37 °C and 900 rpm. Expression cultures were inoculated by addition of 25 µL of overnight culture to 425 µl fresh medium and protein expression was induced by addition of anhydrotetracycline (200 ng/mL) after growth for 2 h at 37 °C and cultivation continued at 20 °C and 900 rpm overnight. Cells were harvested (4 °C, 3220 × *g*, 20 min) and subsequently lysed by addition of 200 µL 25 mM Tris-HCl pH 8.0 containing 0.1% (*v/v*) Triton X-100, 100 µg/mL chicken egg white lysozyme and 0.8 U/mL benzonase (Merck), incubated for 30 min at 20 °C and 600 rpm. Cell debris was sedimented (4 °C, 3220 × *g*, 60 min) and 20 µL cleared lysate was used for determination of enzyme activity in a total reaction volume of 200 µL by measuring the initial rate of absorbance increase at 340 nm for 10 min. Amination activity was measured in the presence of 5 mM 4-phenyl-2-butanone and 0.5 mM NADH in 0.5 M NH_4_Cl/NH_4_OH pH 9.6 and the deamination activity was measured in the presence of 5 mM (*R*)-1-methyl-3-phenylpropylamine and 2 mM NAD^+^ in 100 mM glycine-KOH pH 10.0.

### Enzyme purification and characterization

Expression cultures in 50 mL LB medium supplemented with 100 µg/mL ampicillin were inoculated to an OD_600_ of 0.05 and grown at 37 °C and 200 rpm until an OD_600_ of 0.4–0.8 was reached, at which time protein expression was induced by addition of anhydrotetracycline (200 ng/mL) and cultivation continued overnight at 20 °C and 200 rpm. Cells were pelleted (3220 × *g*, 20 min, 4 °C), lysed in 5 mL lysis buffer for 30 min (1× BugBuster (Merck), 1 µL/mL Lysonase (Merck) in 100 mM glycine-KOH pH 9.0) and cleared (30,000 × *g*, 1 h, 4 °C). Proteins were purified from cleared cell lysate via affinity chromatography using Step-Tactin Sepharose (IBA Lifesciences), according to the manufacturer’s instructions. Purified protein was used for characterization after buffer exchange (PD-10 desalting columns, GE Healthcare) to 100 mM glycine-KOH pH 9.0. Soluble expression was determined by densitometric analysis of SDS-PAGE gels of raw extracts, soluble fraction and the pellet from lysed expression cultures. Steady-state kinetics for deamination was acquired using final concentrations of 0.1 µM pure enzyme, otherwise analogous to the described lysate activity assay. Substrate concentrations ranged from 0 to 12.8 mM *R*-1-methyl-3-phenylpropylamine. Differential scanning fluorimetry with SYPRO orange was performed to determine the melting temperature. A protein concentration of 10 µM combined with 10x SYPRO orange was used in a thermal shift assay. Small scale biotransformations were set up as 500 µl reactions to determine the total turnover number with a final concentration of 0.1 µM pure enzyme and 50 mM 4-phenyl-2-butanone in 1 M NH_4_Cl/NH_4_OH pH 9.6. The cofactor (2 mM NADH) was recycled by addition of 10 mg/mL glucose dehydrogenase (GDH-101) and 100 mM glucose. After 72 h at 30 °C and 800 rpm, reactions were extracted with 1 mL ethyl acetate and analyzed by UHPLC. Measurements were performed on a 1290 Infinity II LC system (Agilent), fitted with a XBridge Phenyl column (4.6×30 mm, Waters) using a water-acetonitrile gradient with 0.1% (*v/v*) trifluoroacetic acid (10% acetonitrile for 30 sec, increase to 90% acetonitrile within 90 s, hold for 30 s) at 20 °C and a flow rate of 0.3 mL/min. 1-methyl-3-phenylpropylamine eluted at 1.43 min and 4-phenyl-2-butanone at 1.93 min and were detected at 210 nm. Conversions were determined from observed peak areas and used for the calculation of total turnover numbers.

### Reporting summary

Further information on research design is available in the Nature Research Reporting Summary linked to this article.

## Supplementary information

Supplementary Information

Description of Additional Supplementary Files

Supplementary Data 1

Supplementary Data 2

Reporting Summary

## Data Availability

Sequencing data generated in this study are deposited in the European Nucleotide Archive under the accession code PRJEB35468. All finalized consensus sequences in FASTA format as well as a data file containing the corresponding mutations and counts in each round are available as Supplementary Data Files. Other data are available from the authors upon request. Source data are provided with this paper.

## Source data

Source Data

## Code availability

All scripts used for the UMIC-seq workflow and an analysis of example data are available at https://github.com/fhlab/UMIC-seq^64^. Scripts to recreate the sequence similarity networks of Fig. 3 and the UMI simulations of Supplementary Fig. 1 are available at https://github.com/fhlab/UMIC-seq/tree/master/figures.
