# Supplementary material for: UMI-linked consensus sequencing enables phylogenetic analysis of directed evolution
Source: Nat Commun. 2020 Nov 26;11:6023. doi: 10.1038/s41467-020-19687-9 (PMC7691348; doi:10.1038/s41467-020-19687-9)
Supplement: Supplementary file 1 — Supplementary Information [file 41467_2020_19687_MOESM1_ESM.pdf]

UMI-linked consensus sequencing enables phylogenetic analysis of directed evolution

Paul Jannis Zurek[1,2], Philipp Knyphausen[1], Katharina Neufeld[1,2], Ahir Pushpanath[2] and Florian Hollfelder[1]*

[1] Department of Biochemistry, University of Cambridge, Cambridge, CB2 1GA, United Kingdom. [2] Johnson Matthey Plc, Cambridge, CB4 0WE, United Kingdom.

*To whom correspondence may be addressed. Email: fh111@cam.ac.uk

Table of Contents

Supplementary Fig. 1: Effect of UMI length and library size on cluster completeness and homogeneity. **S2**

Supplementary Fig. 2: Reduction of error-rates by filtering of mutations and dependence of the error-rate on cluster size. **S3**

Supplementary Fig. 3: Microfluidic screening of AmDHs. **S4**

Supplementary Fig. 4: Devices for absorbance-activated droplet sorting. **S5**

Supplementary Fig. 5: Activity of selected mutations in lysate, relative to the non-mutated AmDH. **S6**

Supplementary Fig. 6: Michaelis-Menten plots for purified AmDH variants. **S7**

Supplementary Fig. 7: Differential scanning fluorimetry for melting point determination. **S8**

Supplementary Note 1: Error-rate calculation from the Sanger sequenced reference set. **S9**

- **Supplementary Fig. S1:** Analysis of UMIs. **S9**

Supplementary Note 2: Calculation of sequencing costs. **S10**

Supplementary Sequence 1: A stabilized AmDH, derived from *Rhodococcus sp.* M4 phenylalanine dehydrogenase (Q59771). **S11**

Supplementary Protocol 1: Library preparation for UMI-linked nanopore sequencing. **S12**

- **Supplementary Table S1:** Primers used for UMIC-seq. **S12**

Supplementary Protocol 2: Generation of accurate consensus sequences. **S16**

- **Supplementary Fig. S2:** Flow chart depicting the bioinformatic workflow to generate consensus sequences from nanopore reads. **S16**

Supplementary Table 1: Primers used to generate variants with IVA cloning. **S19**

Supplementary References **S20**

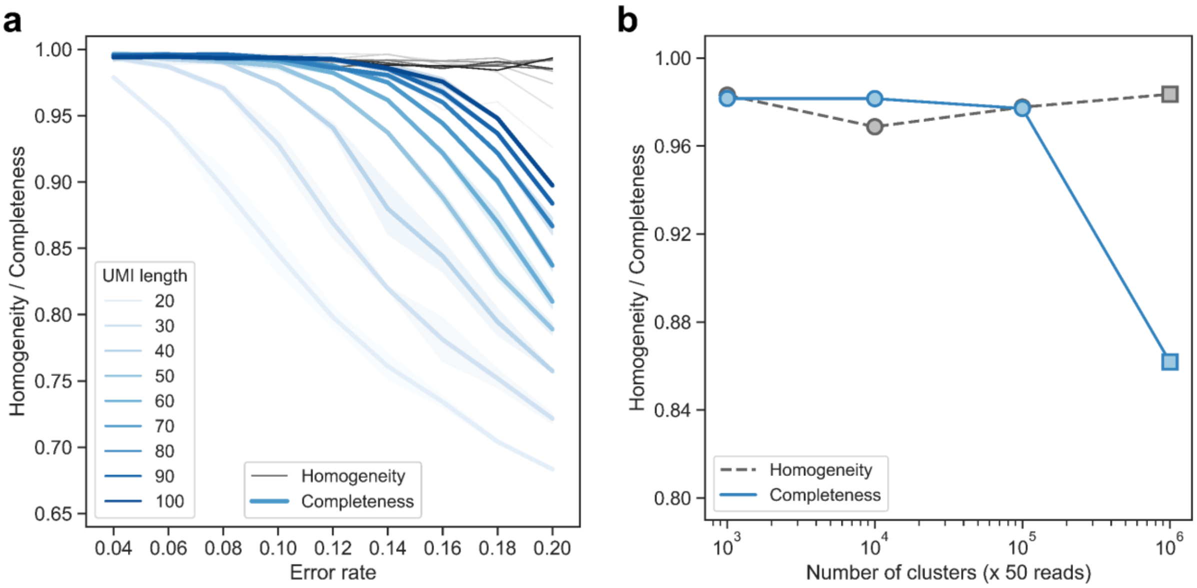

Supplementary Fig. 1: Effect of UMI length and library size on cluster completeness and homogeneity. UMIs were generated as random sequences of different lengths and a simple model of completely random substitution errors was employed to generate erroneous reads of the UMI. All reads were then clustered with the UMIC-seq tools (https://github.com/fhlab/UMIC-seq) and analyzed for cluster homogeneity and completeness. Clustering thresholds were set to keep cluster homogeneity, i.e. the score relating to a cluster containing only members of a single class, as high as possible and over 90%. Completeness values, i.e. the score relating to all members of a single class being assigned to the same cluster, are thus decreased in some conditions. In a real sequencing experiment, high homogeneity would indicate reliable clusters and thus high-quality consensus sequences, while decreased completeness could be counteracted with overall increased sequencing coverage. **(a)** *Influence of UMI length and error rate.* 1000 clusters with an average of 50±5 members each were simulated in independent triplicates to test the effect of UMI length on the clustering efficiency over a range of error rates. UMI lengths of 20 bp to 100 bp were considered and are color coded from light to dark. Grey lines show the cluster homogeneity. Completeness plotted as blue lines with standard deviation. For the final experiment, a UMI length of 50 bp was chosen to achieve a good clustering efficiency (completeness greater than 90% at 14% error rate) at a comparatively low length. **(b)** *Influence of library size.* Clusters of 10^3 to 10^6 UMIs of 50 bp length with an average of 50±5 members each were generated at an error-rate of 0.08±0.01 in independent triplicates. Clustering thresholds were set to maintain high cluster homogeneity. Clustering was performed with the UMIC-seq tools for all cluster sizes (round marker), except for the clustering of the 10^6 UMI dataset (corresponding to 50 x 10^6 erroneous reads). This very large library size required a faster clustering solution and was performed with MMseqs2 Linclust[1] (square marker). While completeness drops to 86% at a library size of 10^6 clusters, homogeneity remains high thus showing applicability of the UMI-clustering approach for sequencing of very large libraries.

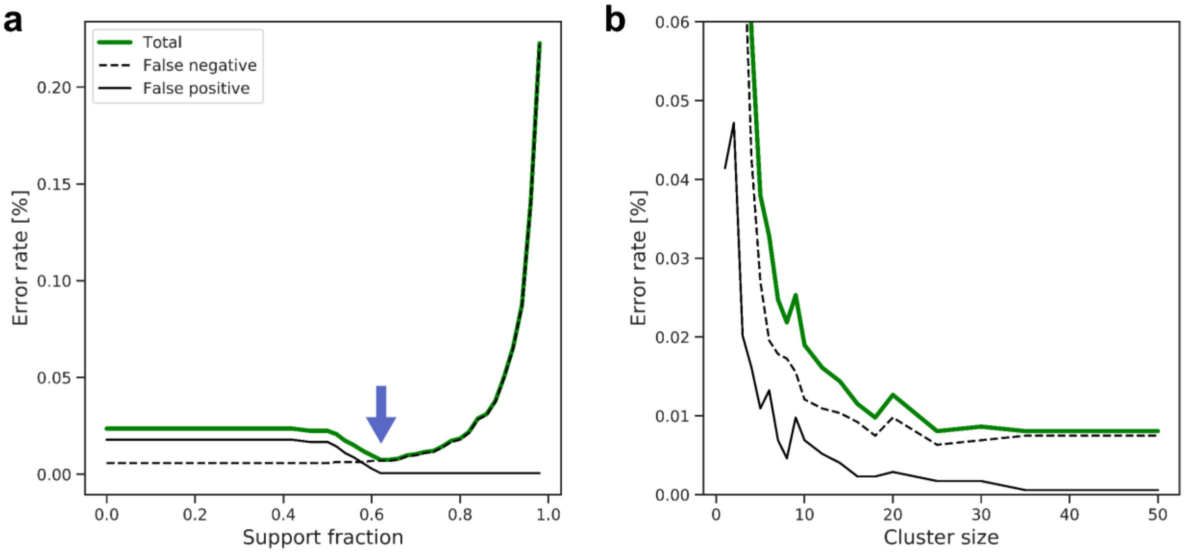

Supplementary Fig. 2: Reduction of error-rates by filtering of mutations and dependence of the error-rate on cluster size. The analysis of error-rates was done with 173 kb of Sanger sequencing data from 180 control reads. These Sanger sequences have a one-to-one match to a UMI and cluster in the nanopore sequencing data. **(a)** *Error-rates are dependent on the support fraction cut-off.* Each mutation identified by nanopolish[2,3] is associated with a support fraction value. Filtering of mutations by support fraction influences the number of false positive and false negative mutations (solid and dashed line, respectively). If mutations with a support fraction greater than 0.6 are accepted, the false positive rate drops without increasing false negative rate (indicated by a blue arrow). **(b)** *Error-rates are dependent on cluster size.* The nanopore clusters corresponding to the Sanger control sequences can be sampled at different depths to infer a dependence of sequencing depth (cluster size) to error-rate. The error-rate stabilizes below 0.01% when clusters contain more than 35 sequences.

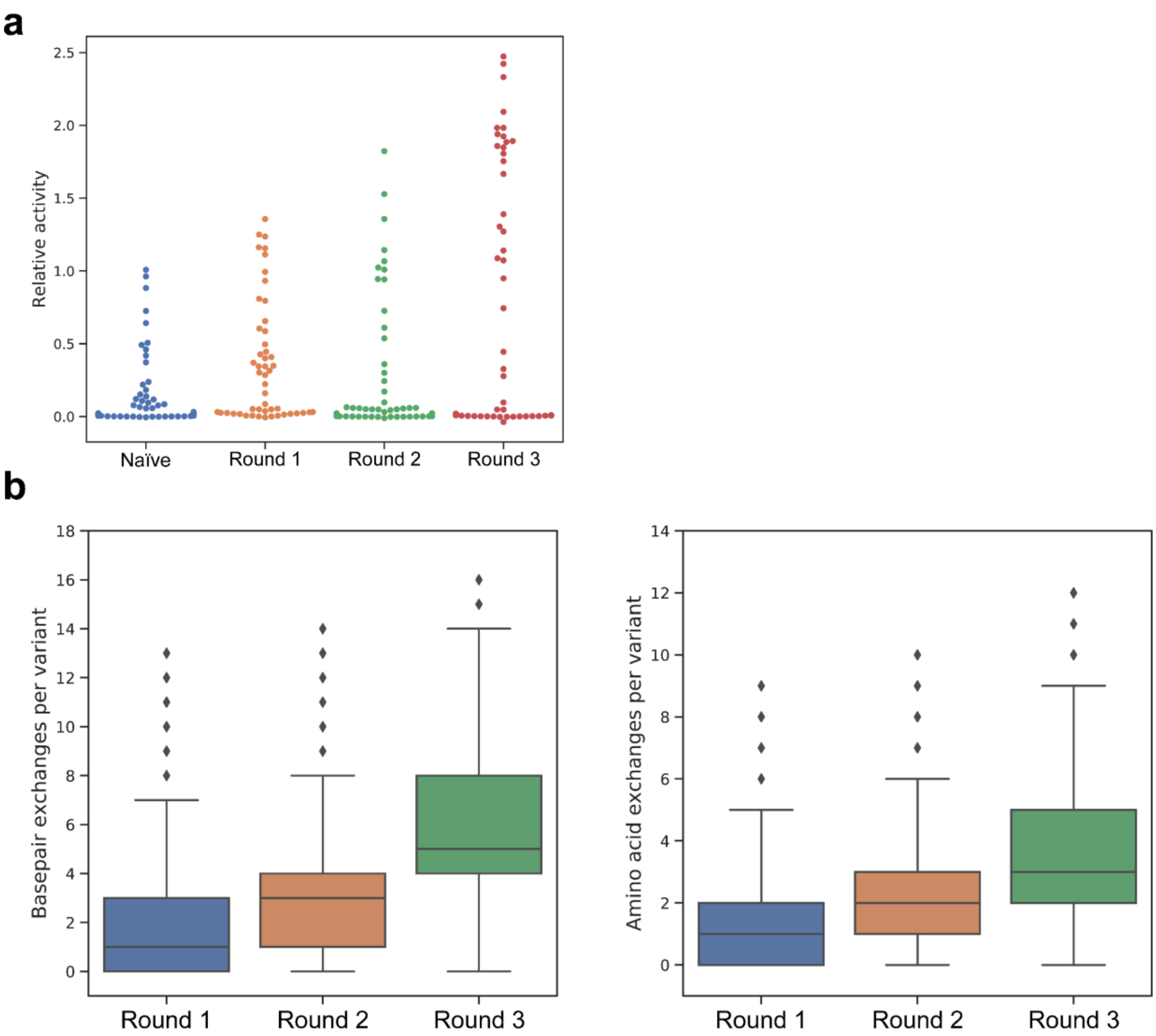

Supplementary Fig. 3: Microfluidic screening of AmDHs. (a) *Validation of sorting success.* The activity of 46 randomly picked variants from the randomized library before sorting (naïve) and after each sorting round was compared to the non-mutated AmDH. The increase in activity over the course of the directed evolution campaign confirms successful enrichment of active variants. **(b)** *Distribution of mutations per variant for each round of directed evolution.* Base pair exchanges (left) and amino acid exchanges (right) per variant shown for each round of evolution. Analyzed n=1665, n=1620 and n=2207 independent reads for each of the rounds, respectively, shown in box-and-whisker plots (box defined by the three quartiles, whiskers as 1.5 IQR of the lower and upper quartile, outliers greater than the whiskers marked independently). The median number of base pair exchanges per variant increases from 1 to 3 to 5 over the three rounds of evolution. This means that most of the variants have more than one mutation raising the issue of epistasis and cooperativity that can only be addressed by full-length sequencing.

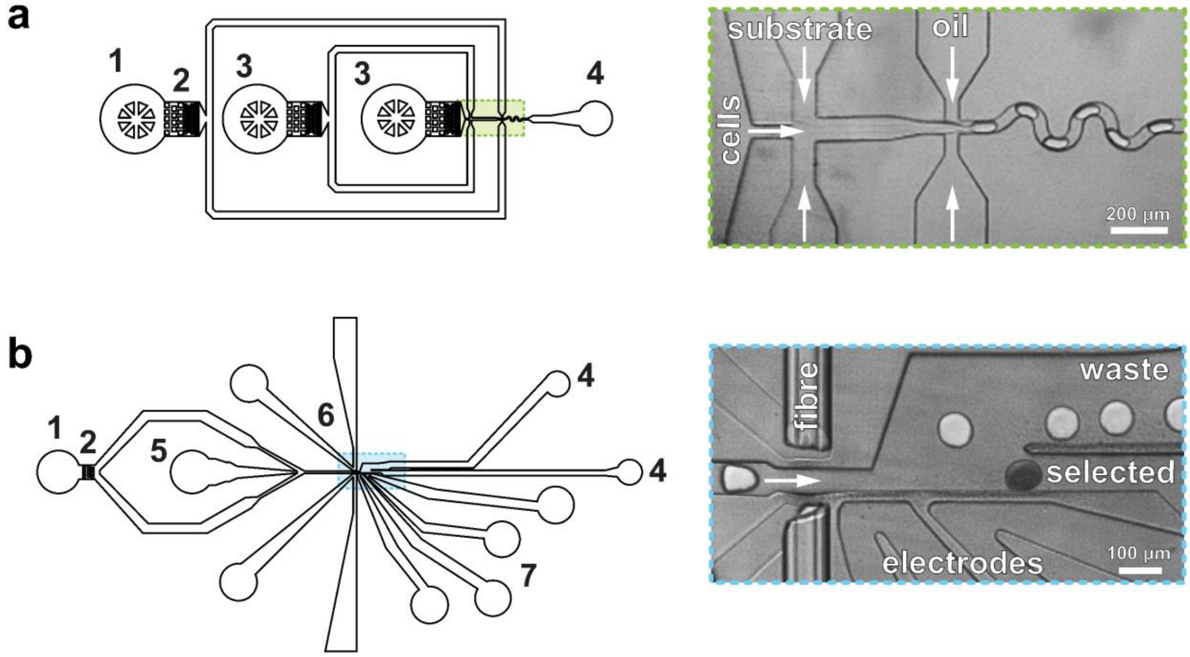

Supplementary Fig. 4: Devices for absorbance-activated droplet sorting. (a) *Flow-focusing device design for droplet generation.* Droplets (280 pL) were generated at >1000 Hz by co-encapsulating a cell suspension with substrate and lysis agent in a fluorinated oil. 1: Inlet for carrier oil. 2: Passive filters. 3: Inlet for reagents. 4: Collection outlet. Chip dimensions at flow-focusing junction: 50 µm width x 80 µm height. **(b)** *Design of the absorbance-activated droplet sorter.* Dimensions as described previously[4]. Optical fibers were inserted into the device to measure the absorbance of each re-injected droplet at >100 Hz. In three independent experiments, 1000 droplets were selected from a total of ~250,000 variants. The still images shown are representative snapshots of droplet formation or sorting taken from videos that record many of such events, typically 1 s showing 100-120 droplets[4]. This is corroborated by 2 min recordings of raw absorbance data, showing the same hit selection frequencies as in the videos. The successful enrichment of active variants in these experiments is shown in Supplementary Fig. 3A, confirming the success of the device operations. Droplets passing the selection threshold were sorted by actuation of electrodes filled with 5 M NaCl solution. False colors are overlaid on droplets to illustrate a sorting event. 5: Inlet for droplets. 6: Channel to fix optical fibers. 7: Electrodes.

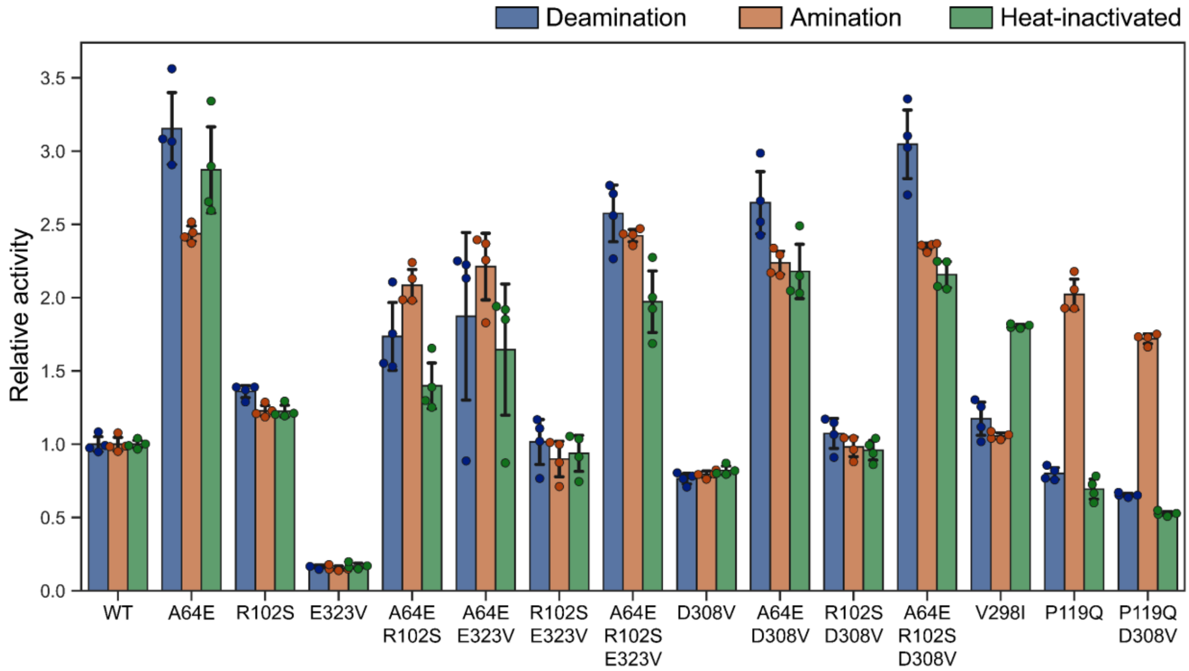

Supplementary Fig. 5: Activity of founder variants in lysate, relative to wild-type AmDH (WT). Individual mutations from founder variants (variants with high frequency and many related sequences, Fig. 3B) and all combinations of their constituent mutations were re-introduced into AmDH wild-type and their effects on activity were tested in lysate (Mean value of n=4 independent biological replicates, error bars show standard deviation). Activity (measured as the initial rate v_o) was tested in both reaction directions (Fig. 2) and after heat-inactivation (10 min 50 °C). *Blue bars:* Deamination activity, 5 mM *R*-1-methyl-3-phenylpropylamine. *Orange bars:* Amination activity, 5 mM 4-phenyl-2-butanone. *Green bars:* Activity after heat-inactivation. Sign epistasis can be identified thanks to the long-read information for example in one of the founder variants (A64E R102S E323V). The mutation E323V individually decreases function drastically to 14.6%-16.8% (95% confidence interval stated, $p = .012$) of the parental AmDH deamination activity. When introduced into the variant A64E R102S it has a beneficial impact, increasing the deamination activity of variant A64E R102S by 121%-187% (95% confidence interval stated, $p = .012$). Gaussian distribution was assumed and ordinary one-way ANOVA ($F(6,21) = 42.6$, $p < .0001$) was performed with post-hoc multiple comparison via Tukey's test. Confidence intervals of mean quotients calculated by the method of EC Fieller[5]. No further beneficial epistatic interactions within the core sets were found but are likely to emerge in further rounds of evolution. Source data are provided as a Source Data file.

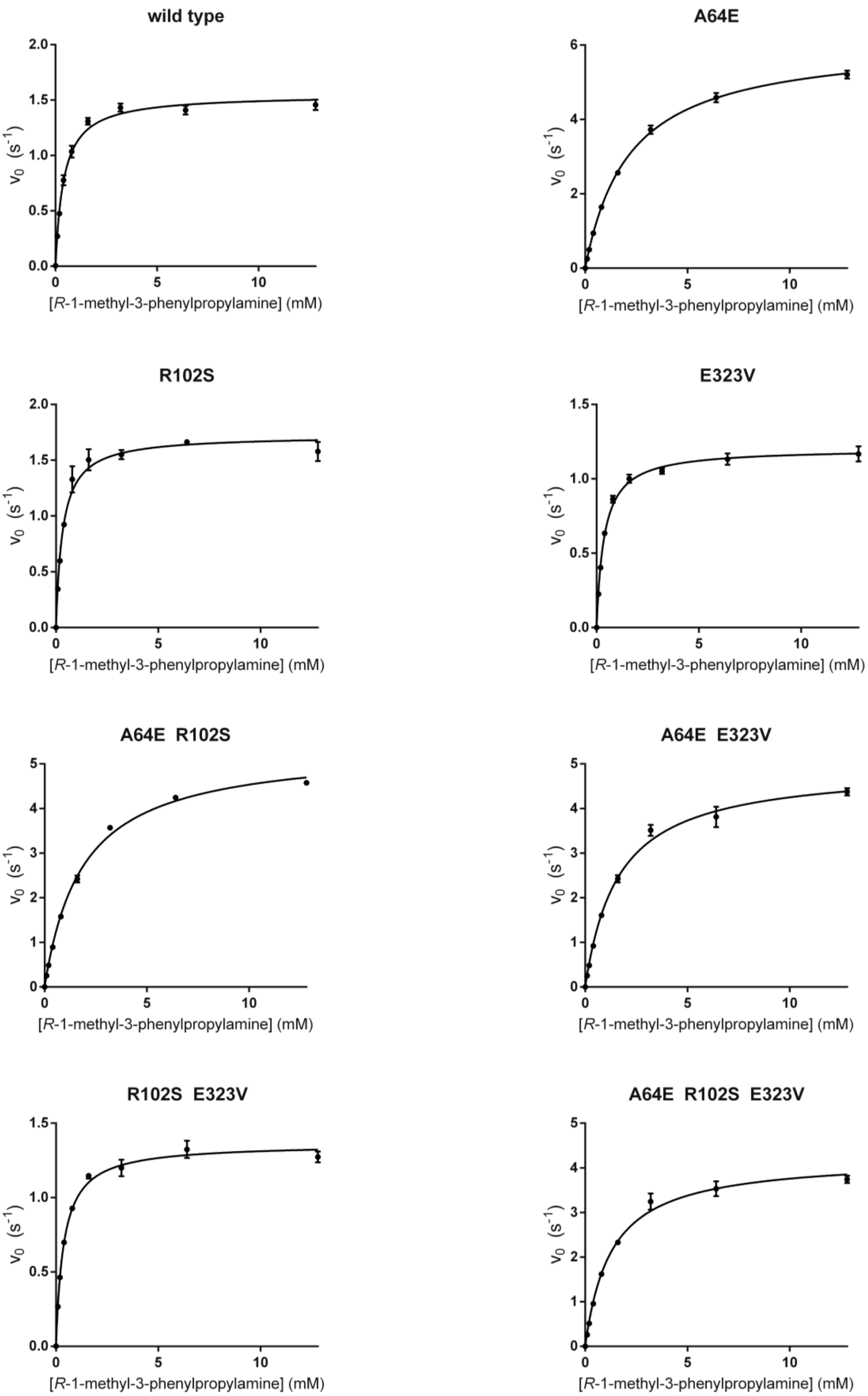

Supplementary Fig. 6: Michaelis-Menten plots for purified AmDH variants. Reaction conditions: Buffer: 100 mM Glycine-KOH pH 10. Co-substrate: 2.5 mM NAD^+. Temperature: 22°C. Substrate: *R*-1-methyl-3-phenylpropylamine 0 mM to 12.8 mM. Measurements in n=3 independent technical replicates, error bars show standard deviation. Source data are provided as a Source Data file.

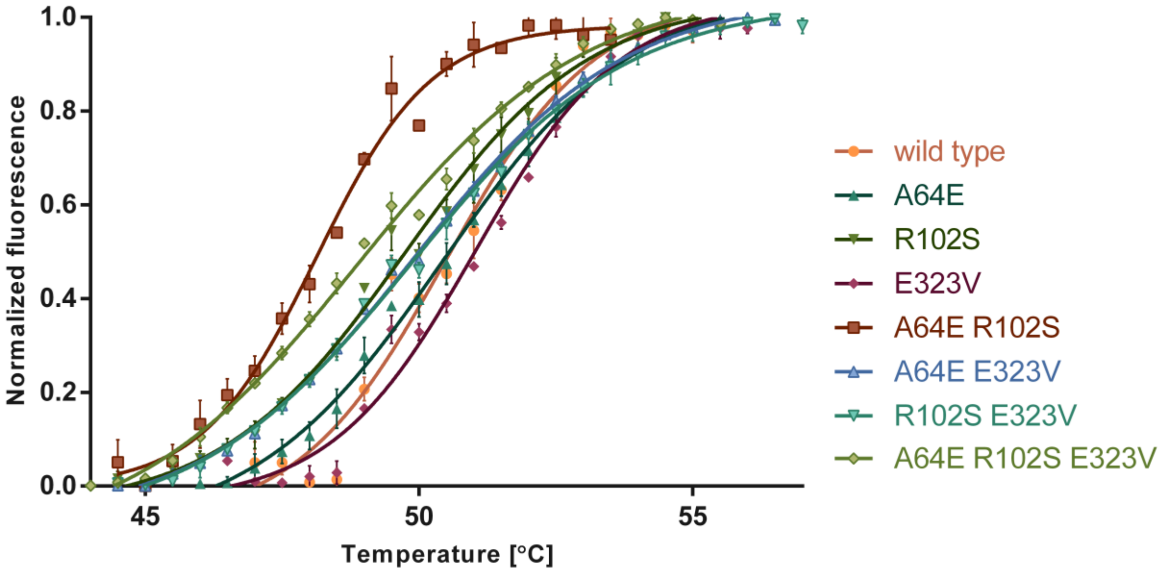

Supplementary Fig. 7: Differential scanning fluorimetry for melting point determination. Determined with 10 µM protein and 10x SYPRO orange in 100 mM Glycine-KOH pH 9. Measurements performed in n=3 independent technical replicates, error bars show standard deviation. Source data are provided as a Source Data file.

Supplementary Note 1: Error-rate calculation from the Sanger sequenced reference set.

The error-rate per base of 0.008% was calculated based on all of the 173 kb in the reference set. A less common but more severe fidelity check, however, would be the error-rate per mutation. For most bases in the dataset the supplied reference sequence is correct, only few true mutations are present. In fact, the Sanger sequenced variants of the reference set have 654 mutations, of which 98% were also identified correctly in the nanopore workflow. Consequently, the mean error rate per mutation for the nanopore consensus sequences was 1.99% for false negatives and 0.16% for false positives. The comparably high rate of false negatives is likely a result of errors in clustering. We observed an AT-homopolymer bias in the UMI sequences (Supplementary Fig. S1), which could lead to some mixed clusters for which no mutations are identified as not all reads support each mutation, resulting in an artificial increase in non-mutated sequences (false negatives). This could be improved in the future with more balanced randomization in the UMI with a higher quality primer or a non-continuous UMI design to break homopolymers, for example repeated stretches of five randomized nucleotides followed by short defined sequences. However, the rate of false positive mutation calls remains very low and an inflation of non-mutated sequences due to few false negatives should not hinder downstream analysis, especially if oversampling on gene variant level is considered.

In the current example, we demonstrate the suitability of the workflow for libraries containing < 17 mutations per gene (Supplementary Fig. 3). It is possible that more mutations cannot be as easily aligned to the wild-type sequence. In this case, the assembly of a reference sequence from the individual reads prior to variant calling may be necessary. Variants with more than 16 mutations and mutations with a nanopolish support fraction lower than 0.6 were discarded. Also, the first and last 50 bp of each read were ignored, because their quality was found to be inferior.

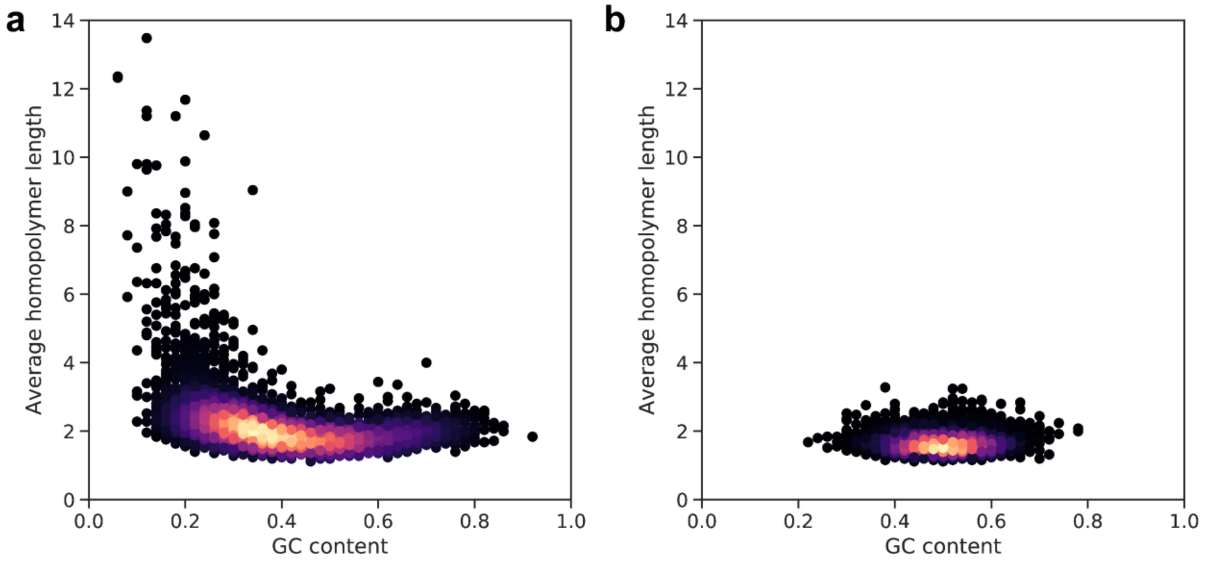

Supplementary Fig. S1: Analysis of UMIs. (a) *Sequenced UMIs show AT-homopolymer bias.* The GC content and homopolymer length in sequenced UMIs (n=6575) is visibly shifted towards AT-rich and homopolymer containing sequences. This bias in UMIs can lead to clustering inefficiencies and could be remedied by changing UMI design to be more intermittent, for example by including fixed bases after every five randomized bases. **(b)** *Random sequences.* An equal number of UMI sequences (n=6575) were randomly generated and analyzed.

Supplementary Note 2: Calculation of sequencing cost.

At the time of writing, one R9.4.1 flow cell for the MinION sequencer from Oxford Nanopore Technologies (ONT) costs 900 USD, with a typical throughput of 8-15 Gb. The library preparation kit, Ligation Sequencing Kit SQK-LSK109, is sold for 599 USD (6 reactions: ~100 USD per reaction) with the required consumables (NEBNext Companion Module E7180S, 24 reactions: ~38 USD per reaction) costing 920 USD. Thus, assuming a read length of 2 kb and an average of 10 Gb sequencing output, a final number of 100,000 accurate consensus sequences can be generated at 50x coverage for around 1050 USD. This corresponds to an approximate cost of 1.1¢ per consensus sequence.

Prices and throughput accessed on 05. Sept 2019 at https://store.nanoporetech.com/flowcells.html and https://www.neb.com/.

Supplementary Sequence 1: A stabilized AmDH, derived from *Rhodococcus sp.* M4 phenylalanine dehydrogenase (Q59771)[4].

```
>AmDH
MGSIDSALNWDGEMTVTRFDAATGAHFVIRIHSTQLGPAAGGTRAWQYSSWADALTDAGRLARAMTYQ
MAVAGLPMGGGKSVIALPAPRHSIDPSTWARILRAHAEMIDSLNGRYWTGPDVNTNSADMDILADETE
FVFGRSPERGGAGSSAFTTALGVFEAMKATVAHRGLGSLDGLTVLVQGLGAVGGSLAKLLAEAGAQLL
VADTDTERVALAVELGHTWVALDDVLSTPCDVFAPCAMGGVITDEVARTLDCKVVCGAAMNVLAHEAA
ADILHARGILYAPDFVANAGGAIHLVGREVLGWSEDQVHERARAIGDTLKEVFEIADKDGVTPDEAAR
ELAERRMREASTTTATA
```

Supplementary Protocol 1: Library preparation for UMI-linked nanopore sequencing.

Notes: Any plasmid can be used as template, as the variants are tagged via PCR. In this case, we used pASK-IBA63b+. After UMI-tagging, it is useful to switch to a different acceptor plasmid that has an alternative selection marker to eliminate the transfer of non-tagged variants. We used pRSFDuet-1 with kanamycin resistance for this purpose. To prepare the sequencing input, the tagged variants are excised from the plasmid with restriction sites that were introduced on the primers. In this protocol, the PCR annealing temperature is given according to the primers used (Supplementary Table S1) and the PCR extension time is given according to the gene length in this study (~1.1 kb). Both parameters can be adjusted to suit other experiments.

Supplementary Table S1: Primers used for UMIC-seq. Important regions are color coded: *Grey:* Homologous region for Gibson assembly. *Red:* UMI. *Blue:* Experiment specific barcode. *Green:* Restriction enzyme sites (*Bam*HI, *Kpn*I). *Yellow:* Spacer sequence. *Black:* Region homologous to the pASK-IBA63b+ plasmid outside of the gene of interest.

Name	Sequence
F_BC1	ACCATCATCACCACAGCCAGGATCC GATAC AAGAAAGTTGTCGGTGTCTTTGTG CTAGAAATAATTTTGTTTAACTTTAAGAAGGAGATATACCATG
F_BC2	ACCATCATCACCACAGCCAGGATCC GATAC TCGATTCCGTTTGTAGTCGTCTGT CTAGAAATAATTTTGTTTAACTTTAAGAAGGAGATATACCATG
F_BC3	ACCATCATCACCACAGCCAGGATCC GATAC GAGTCTTGTGTCCCAGTTACCAGG CTAGAAATAATTTTGTTTAACTTTAAGAAGGAGATATACCATG
R_UMI	GTTTCTTTACCAGACTCGAGGGTACC GATAC NNNNNNNNNNNNNNNNNNNNNNNNN GATAC NNNNNNNNNNNNNNNNNNNNNNNNN GCTCCAAGCGCTCTCGAG
F_gibson	ACCATCATCACCACAGCCAG
R_gibson	GTTTCTTTACCAGACTCGAGGGTAC

Materials:
Zymo Clean & Concentrator-5 (Zymo Research)
High-efficiency electrocompetent *E. coli* cells (e.g. E. cloni 10G ELITE, Lucigen, #60051-1)
GeneJET Plasmid Miniprep Kit (ThermoFischer Scientific)
SQK-LSK109 sequencing kit and flow cell (Oxford Nanopore Technologies, ONT)

Reagents:
Nuclease-free water (Ambion)
Plasmid DNA (Pool of variants in pASK-IBA63b+)
Acceptor plasmid (pRSFDuet-1)
Primers (Supplementary Table S1)
Q5 High-Fidelity 2X Master Mix (NEB)
Gibson Assembly Master Mix (NEB)
FastDigest *Kpn*I and *Bam*HI
Phosphate Buffered Saline (PBS)
AMPure XP SPRI beads (Beckman Coulter)

Protocol:

All steps are performed in parallel for each experiment-specific reaction until sequencing. In this case, the pools of variants from three rounds of evolution are treated in parallel with experiment-specific barcodes.

1) Attaching the UMI and an experiment-specific barcode to the pooled variants

• Prepare UMI tagging reaction in one PCR tube per experiment (e.g. each round of evolution):

Reaction:

25 µl	Q5 High-Fidelity 2X Master Mix
2.5 µl	Forward primer: F_BC
2.5 µl	Reverse primer: R_UMI
500 ng	Template DNA (Variant plasmid pool)
to 50 µl	Nuclease-free water

PCR Program:

98 °C	1 min	
98 °C	10 s	| 2 cycles
60 °C	30 s	|
72 °C	1 min	|
72 °C	5 min	
4 °C	Hold	

• Purify PCR reaction with the Zymo Clean & Concentrator-5 according to the manufacturer's instructions. Elute DNA in 10 µl elution buffer.

• Limited amplification PCR

Reaction:

25 µl	Q5 High-Fidelity 2X Master Mix
2.5 µl	Forward primer: F_gibson
2.5 µl	Reverse primer: R_gibson
250 ng	Template DNA (purified PCR reaction from previous step)
to 50 µl	Nuclease-free water

PCR Program:

98 °C	1 min	
98 °C	10 s	| 15 cycles
60 °C	30 s	|
72 °C	1 min	|
72 °C	5 min	
4 °C	Hold	

• Purify each reaction via agarose gel extraction and clean-up with the Zymo Clean & Concentrator-5. Elute in 10 µl elution buffer.

2) Sub-cloning to restrict molecule diversity via Gibson assembly and transformation.

• Prepare acceptor plasmid via digestion

Reaction:

3 µl	10X FastDigest Green Buffer
1 µg	Acceptor plasmid (pRSFDuet-1)
1 µl	FastDigest *Bam*HI
1 µl	FastDigest *Kpn*I
to 30 µl	Nuclease-free water

PCR Program:

37 °C	60 min
80 °C	5 min

• Purify linearized acceptor plasmid via agarose gel extraction and clean-up with the Zymo Clean & Concentrator-5

• Prepare the Gibson assembly of the tagged variants from step 1 and the acceptor plasmid for each reaction.

Reaction:

100 ng	Linearized acceptor plasmid
100 ng	Tagged variants (~3-fold molar excess over plasmid)
10 µl	Gibson Assembly Master Mix (2X)
to 20 µl	Nuclease-free water

PCR Program:

50°C	60 min

• Purify with Zymo Clean & Concentrator-5. Elute in 10 µl nuclease-free water.

• Transform 25 µl electrocompetent E. cloni 10G ELITE with 5 µl of the purified Gibson assembly for each reaction. Plate a dilution series.

3) Isolation of linear, amplified and tagged molecules

• Select number of colonies:
This determines the number of final consensus sequences that will be generated. Here, 500-1000 variants were obtained during each round of evolution. To achieve at least 3-fold oversampling, 3000 colonies were selected.

• Scrape the selected number of colonies off the transformation plates with the help of ~2 ml PBS, pellet the cells and perform plasmid isolation (GeneJET Miniprep Kit).

• Digest the isolated plasmids to receive the tagged variant sequences for each reaction

Reaction:

5 µl	10X FastDigest Green Buffer
3 µg	Plasmid isolation from the previous step
2 µl	FastDigest *Bam*HI

2 µl	FastDigest *Kpn*I
to 50 µl	Nuclease-free water

PCR Program:

37 °C	60 min
80 °C	5 min

• Purify the tagged insert via agarose gel extraction and clean-up with the Zymo Clean & Concentrator-5. Elute in 100 µl elution buffer.

• Purify again with AMPure XP SPRI beads. Elute in 25 µl nuclease-free water.

4) Nanopore sequencing

• Pool purified tagged fragments equimolarly per barcoded-reaction to a final content of 200 fmol DNA in 47 µl nuclease-free water.

• Follow instructions for further library preparation and sequencing with ONT's Ligation Sequencing Kit (SQK-LSK109).

• Run the sequencing until 100X* expected coverage is reached. Here, 3000 colonies were selected in three barcoded experiments, which thus corresponds to 9000 unique fragments after isolation. The tagged fragment length is ~1250 bp, thus sequencing is run until at least 9000 * 100 = 900,000 reads or 9000 * 100 * 1250 ≈ 1.1 Gb are sequenced.
*Note: This experiment (Supplementary Fig. 2) shows that more than 35X coverage would be sufficient.

Supplementary Protocol 2: Generation of accurate consensus sequences.

Notes: Tools made for this study are made available as Python scripts, hosted on GitHub (https://github.com/fhlab/UMIC-seq). All steps of the bioinformatic analysis workflow are illustrated in Supplementary Fig. S3. Additionally, an example is provided on GitHub, showing the core steps based on a small dataset in detail.

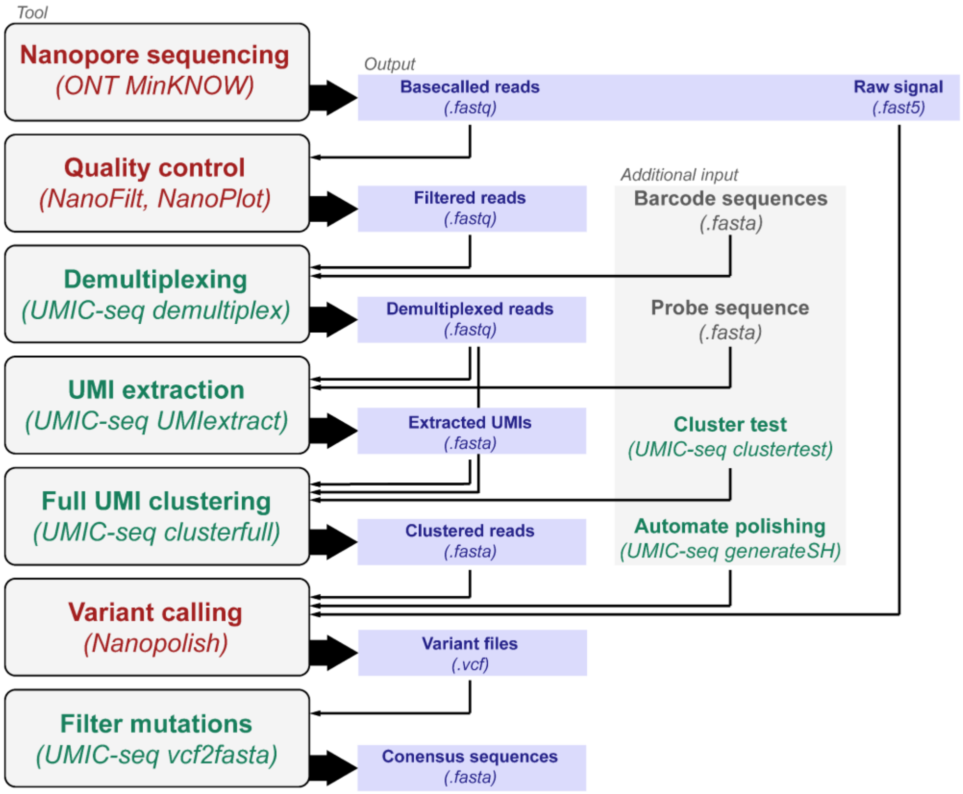

Supplementary Fig. S2: Flow chart depicting the bioinformatic workflow to generate consensus sequences from nanopore reads. Utilized tools are categorized into tools supplied by the UMIC-seq scripts from this study (green text) and external tools (red text). External tools are: MinKNOW from Oxford Nanopore Technologies, NanoFilt[6] and Nanopolish[2,3]. Additional inputs: List of barcode sequences that were used for multiplexing. A probe sequence that is a short constant region adjacent to the UMI. A suitable threshold value for clustering, which can be approximated with the *cluster test* tool. A script to automate polishing can be generated by the *generateSH* tool.

Software requirements:

** installable via the package manager conda*

Unix-based operating system: MacOS, Windows Subsystem for Linux, Ubuntu or similar.

Python* (version > 3.6)

Python packages*

scikit-bio, scikit-allel, biopython

Nanopolish* (version > 10.1)

NanoPlot and NanoFilt*

Porechop*

Porechop can be used to demultiplex reads. As it is no longer officially maintained, demultiplexing was also incorporated into the UMIC-seq scripts.

Requisites:

- Raw and basecalled nanopore reads *(fast5 / fastq)*
- List of barcodes for demultiplexing *(fasta)*
- Probe sequence for UMI extraction *(fasta)*

Steps:
Sequencing and base calling are performed within the MinION software, as explained in the sequencing kit's documentation. The following steps are assuming a sequencing run and basecalling were successfully performed. Some settings, such as the length filtering, have to be adjusted from experiment to experiment. Here, the expected read length was ~1250 bp.

1) Quality control and read filtering

• *NanoPlot:* Plot sequencing metrics for quality control: Visualize read quality and length distribution to find suitable thresholds for filtering.

• *NanoFilt:* Filter sequences based on length and average read quality using NanoFilt: In our case, the quality filtering was stringent – approximately 25% of all reads were discarded during filtering.

2) Demultiplex reads belonging to different experiments

• *Porechop:* Demultiplex the experiment specific barcodes, here to separate the three rounds of evolution. The barcode sequences were taken from the PCR Barcoding Expansion Pack 1-96 (EXP-PBC096).

• *UMIC-seq demultiplex:* As porechop is officially unsupported as of October 2018, demultiplexing was integrated into a helper script. A list of barcodes for demultiplexing must be supplied *(fasta)*, as well as the basecalled reads *(fastq)* and alignment thresholds.

3) Extraction and clustering of UMIs

• *UMIC-seq UMIextract:* To extract the UMI, a probe sequence must be supplied (*fasta*). This probe sequence should be a short constant region (e.g. 50 bp) next to the UMI, i.e. a part of the reference gene. Extraction will copy the sequence next to the probe into a new file. The following steps are run individually for each of the demultiplexed experiments.

• *UMIC-seq clustertest:* UMI clustering threshold approximation: To estimate a suitable clustering threshold, a threshold approximation can be run. The thresholds to test can be specified, e.g. as 20 70 10, which will sample thresholds from 20 to 70 with a step width of 10. The script will output similarity histograms and a clustering test plot. In the similarity histogram, a randomly chosen UMI is aligned to all other UMI sequences and the resulting alignment scores are plotted as a histogram. Here, a lot of low alignment scores as well as few sequences with high alignment scores (a potential cluster) are expected. A suitable clustering threshold separates the two. The clustering test plot will provide clustering information (cluster size and similarity of sequences in a cluster) for clusters with each of

the sampled thresholds. A suitable threshold is found when both metrics begin to saturate. In our experience, the clustering threshold will be close to the length of the UMI.

• *UMIC-seq clusterfull:* Full clustering is performed with the previously determined threshold. The size threshold specifies the minimal size of a cluster to be written to file.

3) Sequence polishing and calling of mutations with Nanopolish

• Each cluster file can now be used for signal level analysis with Nanopolish. Nanopolish will be run on each cluster file to generate the mutations of that cluster (*.vcf*). See the nanopolish documentation for details. Alternatively, a quicker but potentially less accurate consensus generation with medaka can be run similarly to nanopolish.

• *UMIC-seq_helper generateSH:* To automate the execution of nanopolish on each cluster file, the UMIC-seq_helper script can generate a shell script performing the command on all cluster files. A list of cluster filenames needs to be provided. The commands that should be run on each cluster are provided as a text file, with a keyword to be replaced by each cluster file name.

• *UMIC-seq_helper vcf2fasta:* To combine all mutations in all the individual vcf files into full length sequences in one multi-fasta file, the helper script can be run again. Additionally, filtering of mutations by support fraction can be performed.

Supplementary Table 1: Primers used to generate variants with IVA cloning. Founder variants and the combination of their constituent mutations were re-introduced to the wild-type sequence by IVA cloning[7], as described in the Materials and methods. Color code: *Grey:* Homologous recombination region. *Red:* Mutation. *Green:* Additional template binding.

Name	Sequence
A64E-f	GGTCGTCTGGCACGTGAAATGACCTATCAGATGGCAGTTGCAG
A64E-r	ACGTGCCAGACGACCTGCATC
R102S-f	CCTGGGCACGTATTCTGAGTGCACATGCAGAAATGATTGATAGCCTGAATG
R102S-r	CAGAATACGTGCCCAGGTGCTCG
P119Q-f	GGTCGTTATTGGACAGGTCAGGATGTTAATACCAATAGCGCAGATATGGATATTCTG
P119Q-r	ACCTGTCCAATAACGACCATTCAGGC
V298I-f	GGTGGTGCAATTCATCTGATTGGTCGTGAAGTTTTAGGTTGGAGCG
V298I-r	CAGATGAATTGCACCACCGGCATTTG
D308V-f	GTTTTAGGTTGGAGCGAAGTTCAGGTGCATGAACGTGCCCG
D308V-r	TTCGCTCCAACCTAAAACTTCACGAC
E323V-f	CAATTGGTGATACCCTGAAAGTAGTTTTTGAGATCGCAGATAAAGATGGTGTTACAC
E323V-r	TTTCAGGGTATCACCAATTGCACGG

Supplementary references:

1. Steinegger, M. & Söding, J. Clustering huge protein sequence sets in linear time. *Nat. Commun.* **9**, 1–8 (2018).
2. Loman, N. J., Quick, J. & Simpson, J. T. A complete bacterial genome assembled de novo using only nanopore sequencing data. *Nat. Methods* **12**, 733–735 (2015).
3. Quick, J. *et al.* Real-time, portable genome sequencing for Ebola surveillance. *Nature* **530**, 228–232 (2016).
4. Gielen, F. *et al.* Ultrahigh-throughput–directed enzyme evolution by absorbance-activated droplet sorting (AADS). *Proc. Natl. Acad. Sci.* **113**, E7383–E7389 (2016).
5. Fieller, E. C. The Biological Standardization of Insulin. *Suppl. J. R. Stat. Soc.* **7**, 1–64 (1940).
6. De Coster, W. *et al.* NanoPack: visualizing and processing long-read sequencing data. *Bioinformatics* **34**, 2666–2669 (2018).
7. García-Nafría, J., Watson, J. F. & Greger, I. H. IVA cloning: A single-tube universal cloning system exploiting bacterial In Vivo Assembly. *Sci. Rep.* **6**, 27459 (2016).
