# Supplementary material for: UMI-linked consensus sequencing enables phylogenetic analysis of directed evolution
Source: Nat Commun. 2020 Nov 26;11:6023. doi: 10.1038/s41467-020-19687-9 (PMC7691348; doi:10.1038/s41467-020-19687-9)
Supplement: Supplementary file 2 — Description of Additional Supplementary Files [file 41467_2020_19687_MOESM2_ESM.pdf]

Title: Supplementary Data 1:
Description: Final consensus sequences in FASTA format.

Title: Supplementary Data 2:
Description: Identified mutations with read counts per round of directed evolution.
