# Supplementary material for: UMI-linked consensus sequencing enables phylogenetic analysis of directed evolution
Source: Nat Commun. 2020 Nov 26;11:6023. doi: 10.1038/s41467-020-19687-9 (PMC7691348; doi:10.1038/s41467-020-19687-9)
Supplement: Supplementary file 5 — Reporting Summary [file 41467_2020_19687_MOESM5_ESM.pdf]

nature research

Corresponding author(s): F Hollfelder

Last updated by author(s): Sep 28, 2020

Reporting Summary

Nature Research wishes to improve the reproducibility of the work that we publish. This form provides structure for consistency and transparency in reporting. For further information on Nature Research policies, see our Editorial Policies and the Editorial Policy Checklist.

Statistics

For all statistical analyses, confirm that the following items are present in the figure legend, table legend, main text, or Methods section.

n/a	Confirmed	
☐	☒	The exact sample size (*n*) for each experimental group/condition, given as a discrete number and unit of measurement
☐	☒	A statement on whether measurements were taken from distinct samples or whether the same sample was measured repeatedly
☐	☒	The statistical test(s) used AND whether they are one- or two-sided<br>*Only common tests should be described solely by name; describe more complex techniques in the Methods section.*
☒	☐	A description of all covariates tested
☐	☒	A description of any assumptions or corrections, such as tests of normality and adjustment for multiple comparisons
☐	☒	A full description of the statistical parameters including central tendency (e.g. means) or other basic estimates (e.g. regression coefficient) AND variation (e.g. standard deviation) or associated estimates of uncertainty (e.g. confidence intervals)
☐	☒	For null hypothesis testing, the test statistic (e.g. *F*, *t*, *r*) with confidence intervals, effect sizes, degrees of freedom and *P* value noted<br>*Give P values as exact values whenever suitable.*
☒	☐	For Bayesian analysis, information on the choice of priors and Markov chain Monte Carlo settings
☒	☐	For hierarchical and complex designs, identification of the appropriate level for tests and full reporting of outcomes
☒	☐	Estimates of effect sizes (e.g. Cohen's *d*, Pearson's *r*), indicating how they were calculated

Our web collection on statistics for biologists contains articles on many of the points above.

Software and code

Policy information about availability of computer code

Data collection	Nanopore sequencing reads were acquired using MinKNOW for MinION (v2.1, Oxford Nanopore Technologies), containig MinKNOW Core v1.14.1.
Data analysis	All scripts used in the UMIC-seq workflow and an analysis of example data are available at https://github.com/fhlab/UMIC-seq. Scripts to recreate the sequence similarity networks of Fig. 3 and the UMI simulations of Supplementary Fig. 1 are available at https://github.com/fhlab/UMIC-seq/tree/master/figures. The scripts use Python v.3.7 or later with biopython (v.1.74), scikit-bio (v.0.5.5) and scikit-allel (v.1.2.1). Consensus sequences were generated with Nanopolish (v.10.1).

For manuscripts utilizing custom algorithms or software that are central to the research but not yet described in published literature, software must be made available to editors and reviewers. We strongly encourage code deposition in a community repository (e.g. GitHub). See the Nature Research guidelines for submitting code & software for further information.

Data

Policy information about availability of data

All manuscripts must include a data availability statement. This statement should provide the following information, where applicable:

- Accession codes, unique identifiers, or web links for publicly available datasets
- A list of figures that have associated raw data
- A description of any restrictions on data availability

Sequencing data generated in this study is deposited in the European Nucleotide Archive under the accession code PRJEB35468 [https://www.ebi.ac.uk/ena/browser/view/PRJEB35468]. All finalized consensus sequences in FASTA format as well as a data file containing the corresponding mutations and counts in each round are available as Supplementary Data Files. Source data are provided with this paper. Other data are available from the authors upon request.

Field-specific reporting

Please select the one below that is the best fit for your research. If you are not sure, read the appropriate sections before making your selection.

[x] Life sciences   [ ] Behavioural & social sciences   [ ] Ecological, evolutionary & environmental sciences

For a reference copy of the document with all sections, see nature.com/documents/nr-reporting-summary-flat.pdf

Life sciences study design

All studies must disclose on these points even when the disclosure is negative.

Sample size	A sample size of 4 independent biological E. coli replicates was used for lysate activity assays. The expression in E. coli is generally highly reproducible so that 4 samples ensured a good reflection of variant activity. Characterization of purified enzyme variants was confirmed in 3 technical replicates with independent measurements. Three replicates allowed a good estimation of the very small errors mainly due to pipetting.
Data exclusions	In all nanopore sequencing experiments, reads of low quality (average quality score < 10) and consensus sequences with > 16 mutations were excluded during quality control.
Replication	Lysate activities were confirmed in 4 independent biological replicates. Kinetic characterization was confirmed in 3 technical replicates. All replicates demonstrated high degrees of reproducibility in each experiment. Absorbance activated droplet sorting was performed three times, each round of selection showing successful enrichment, confirming its reliability.
Randomization	Randomization was not relevant to this study as samples were not grouped for experiments or analyses.
Blinding	Blinding was not relevant to this study as samples were not grouped for experiments or analyses.

Reporting for specific materials, systems and methods

We require information from authors about some types of materials, experimental systems and methods used in many studies. Here, indicate whether each material, system or method listed is relevant to your study. If you are not sure if a list item applies to your research, read the appropriate section before selecting a response.

Materials & experimental systems

n/a	Involved in the study
[x]	[ ] Antibodies
[x]	[ ] Eukaryotic cell lines
[x]	[ ] Palaeontology and archaeology
[x]	[ ] Animals and other organisms
[x]	[ ] Human research participants
[x]	[ ] Clinical data
[x]	[ ] Dual use research of concern

Methods

n/a	Involved in the study
[x]	[ ] ChIP-seq
[x]	[ ] Flow cytometry
[x]	[ ] MRI-based neuroimaging
